# Supplementary material for: Dual RNA-seq of Orientia tsutsugamushi informs on host-pathogen interactions for this neglected intracellular human pathogen
Source: Nat Commun. 2020 Jul 3;11:3363. doi: 10.1038/s41467-020-17094-8 (PMC7335160; doi:10.1038/s41467-020-17094-8)
Supplement: Supplementary file 1 — Supplementary Information [file 41467_2020_17094_MOESM1_ESM.pdf]

SUPPLEMENTARY INFORMATION

Dual RNA-seq of *Orientia tsutsugamushi* informs on host-pathogen interactions for this neglected intracellular human pathogen

Mika-Gospodorz et al.

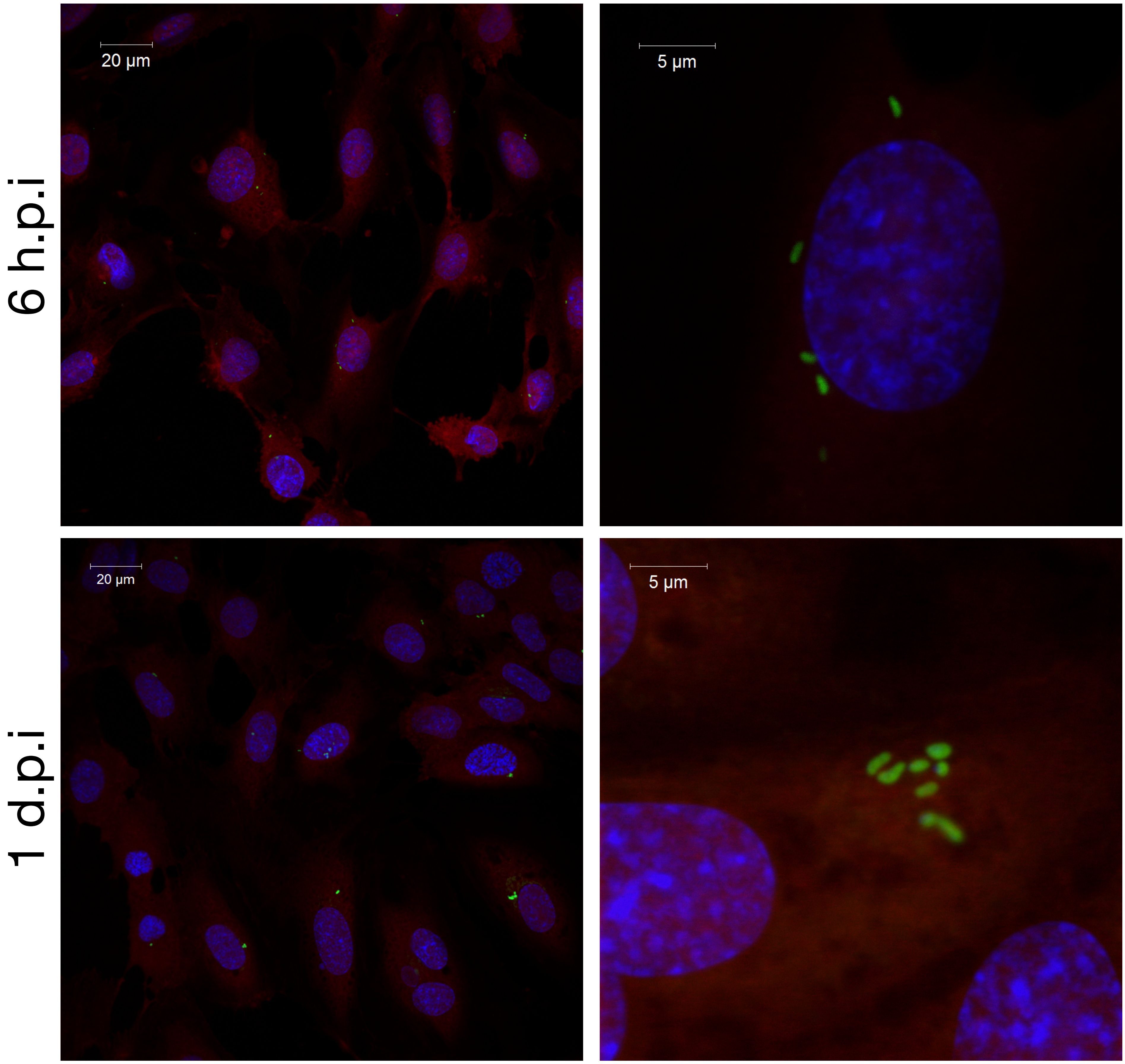

Supplementary Figure 1. Ot_Karp time course. Confocal microscopy images of Karp strain Ot bacteria in HUVEC cells at 0-7 days post infection. Two representative images are shown at each time point. Blue = DAPI (DNA), Red = Evans blue (host cells), green = Ot labelled with Alexa488-click-methionine. Representative images taken from more than ten images of each time point.

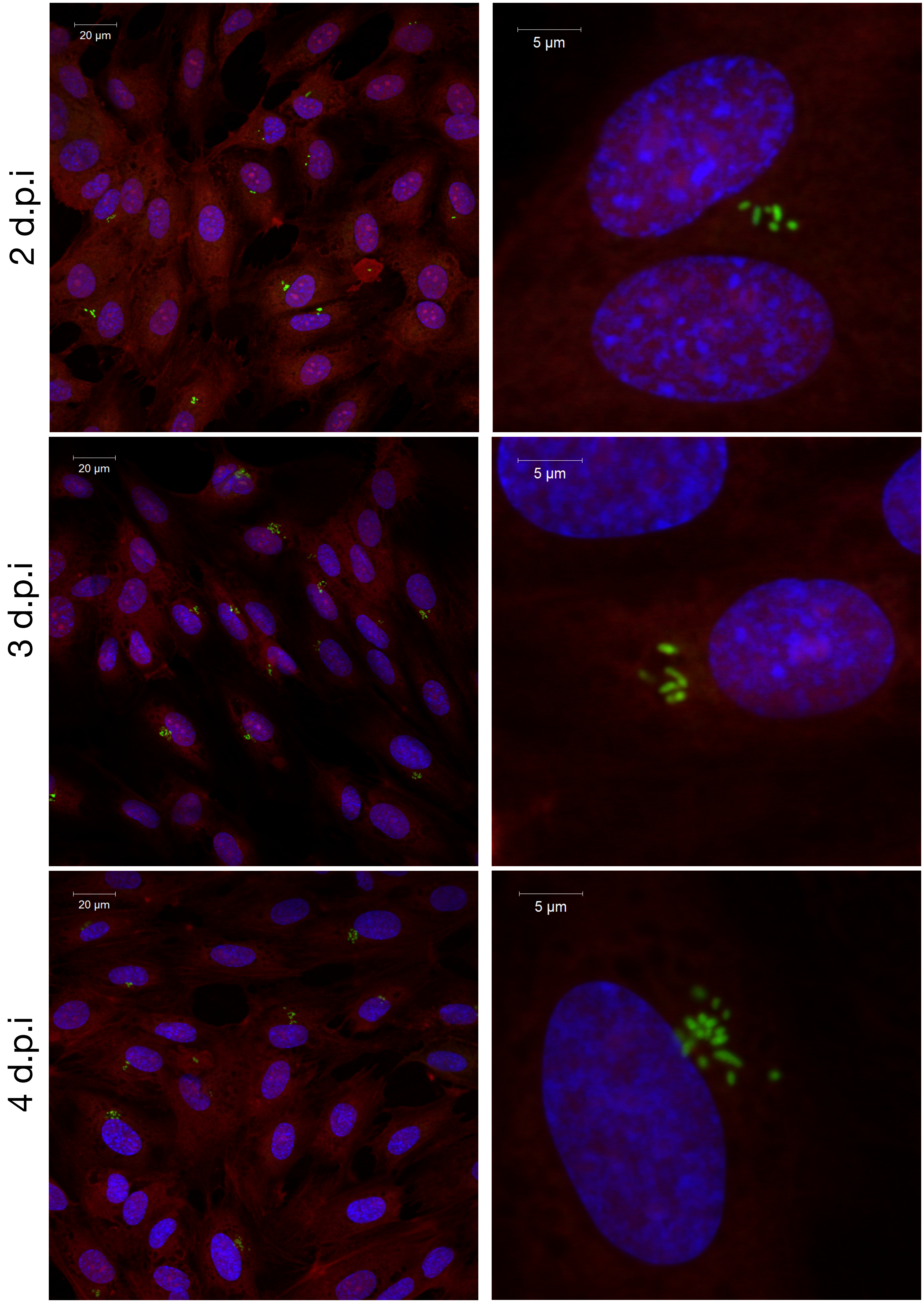

Supplementary Figure 1 continued

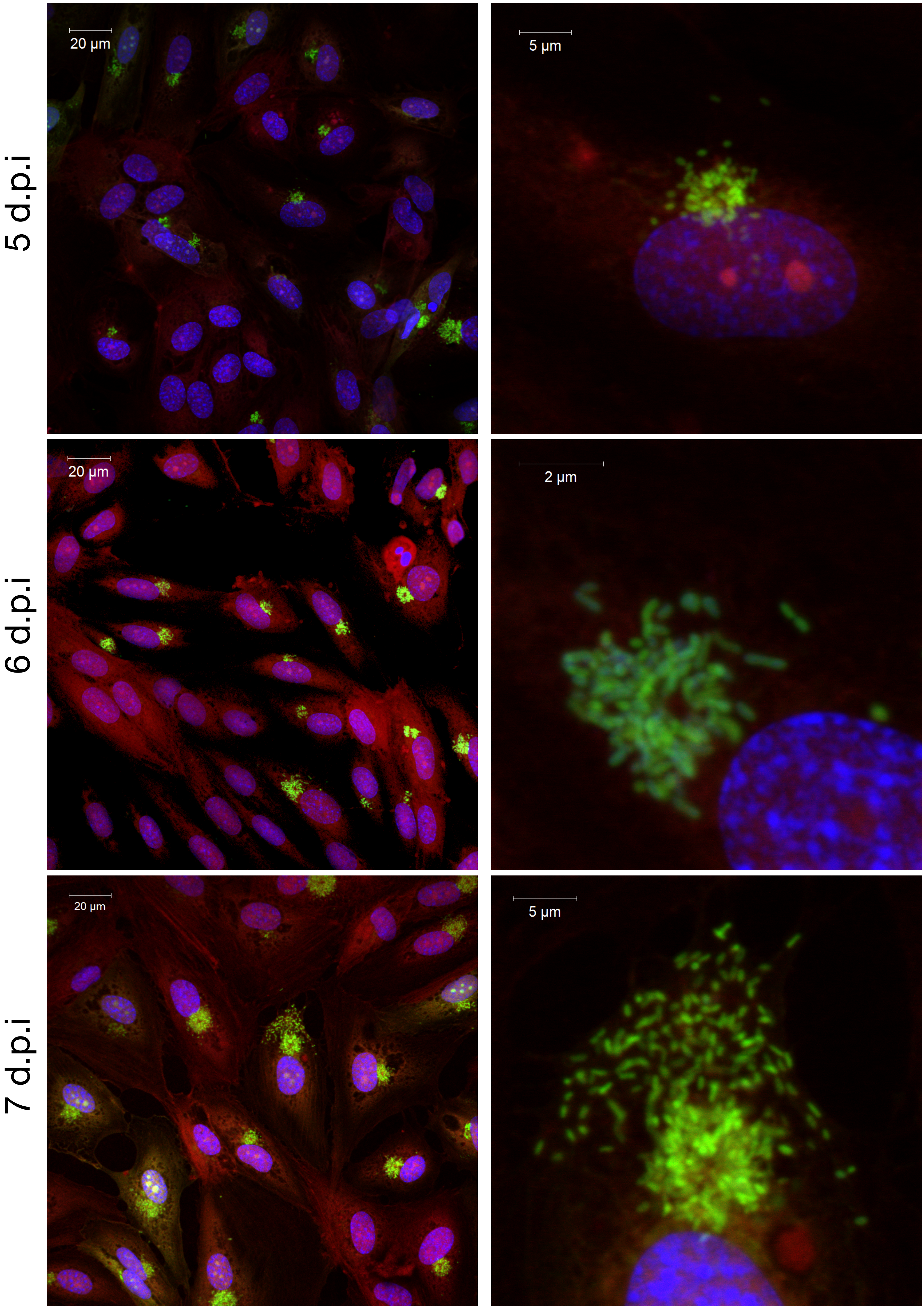

Supplementary Figure 1 continued

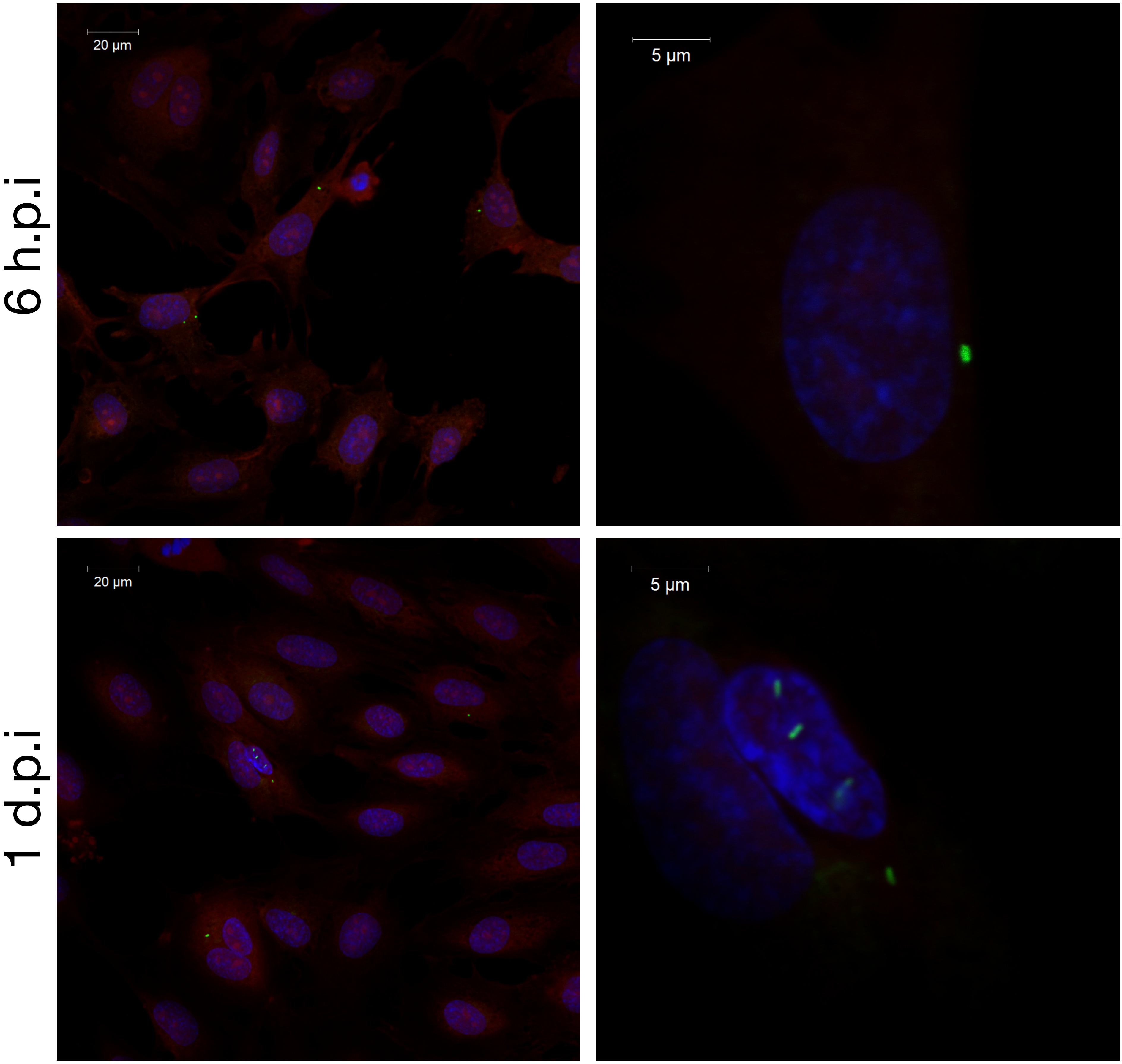

Supplementary Figure 2. Ot_UT176 time course. Confocal microscopy images of Ot_UT176 bacteria in HUVEC cells at 0-7 days post infection. Two representative images are shown at each time point. Blue = DAPI (DNA), Red = Evans blue (host cells), green = Ot labelled with Alexa488-click-methionine. Representative images taken from more than ten images of each time point.

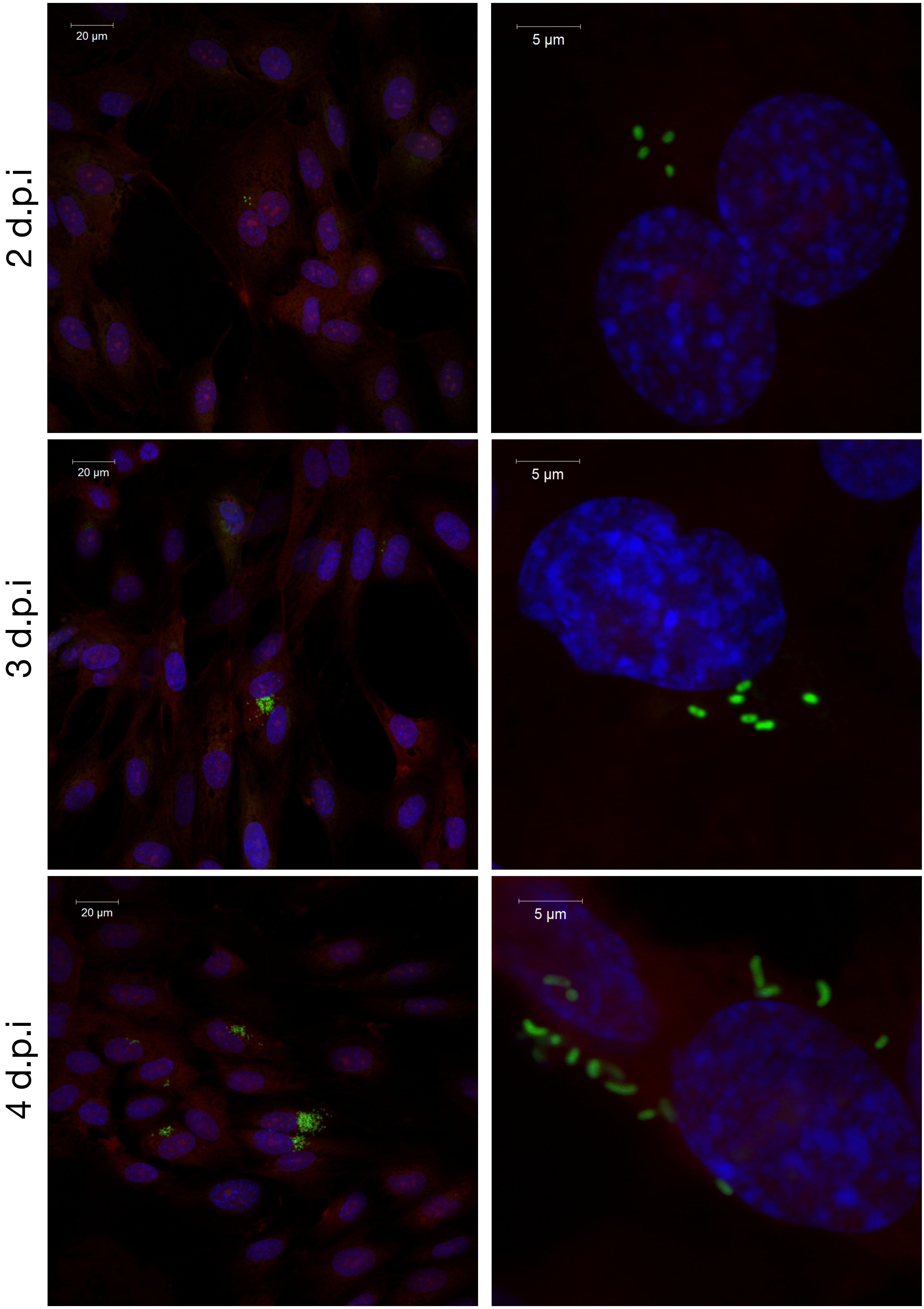

Supplementary Figure 2. continued

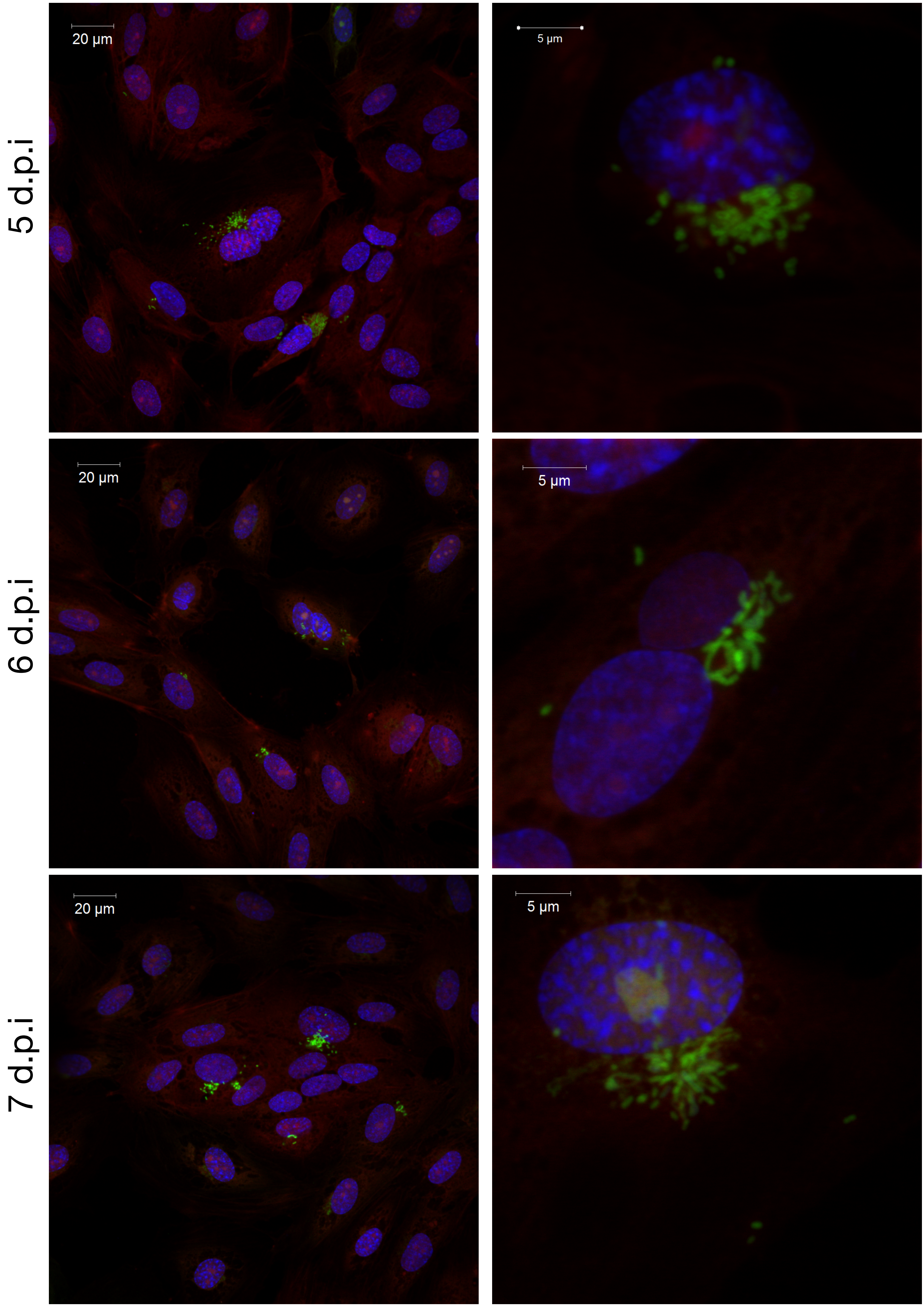

Supplementary Figure 2. continued

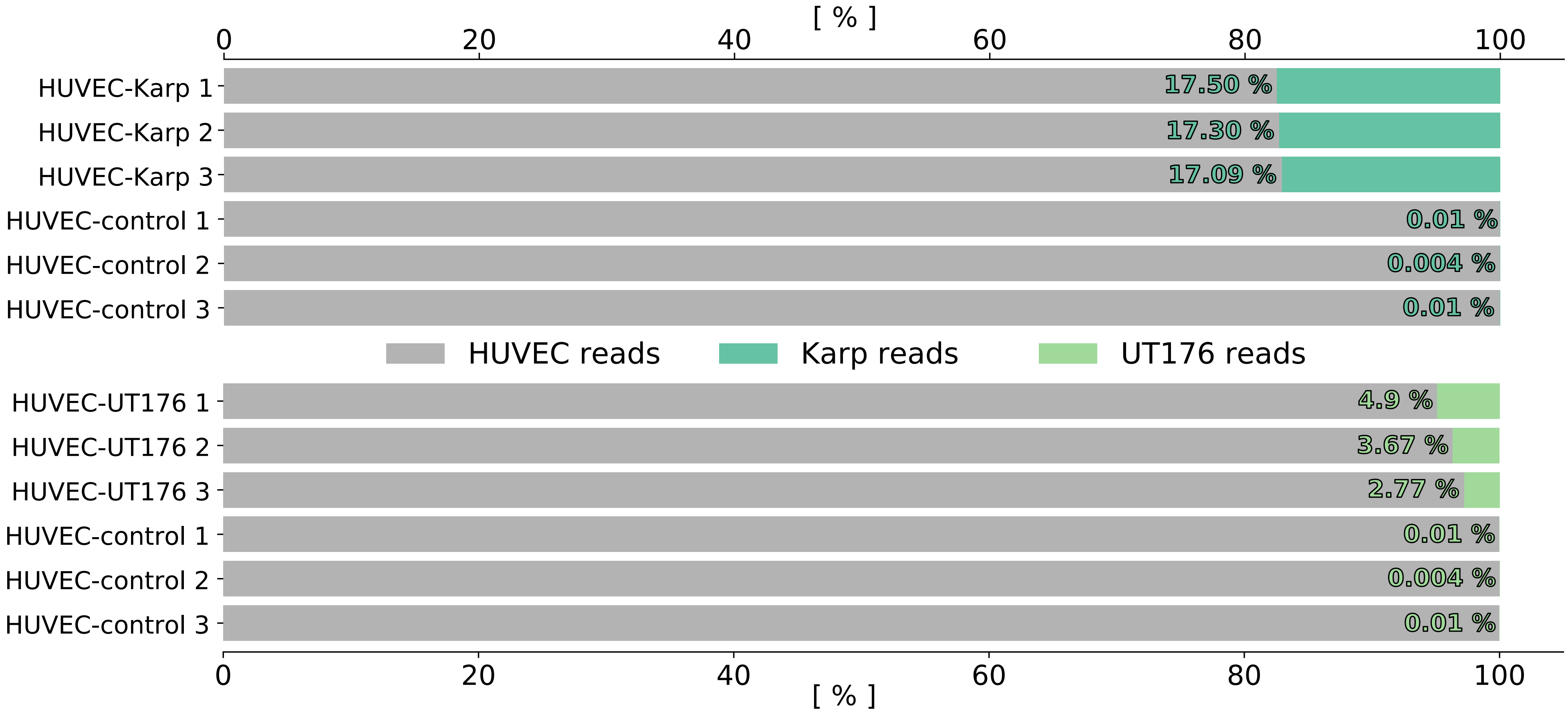

Supplementary Figure 3. RNA mapping statistics showing ratio of host and Ot RNA in each individual sample.

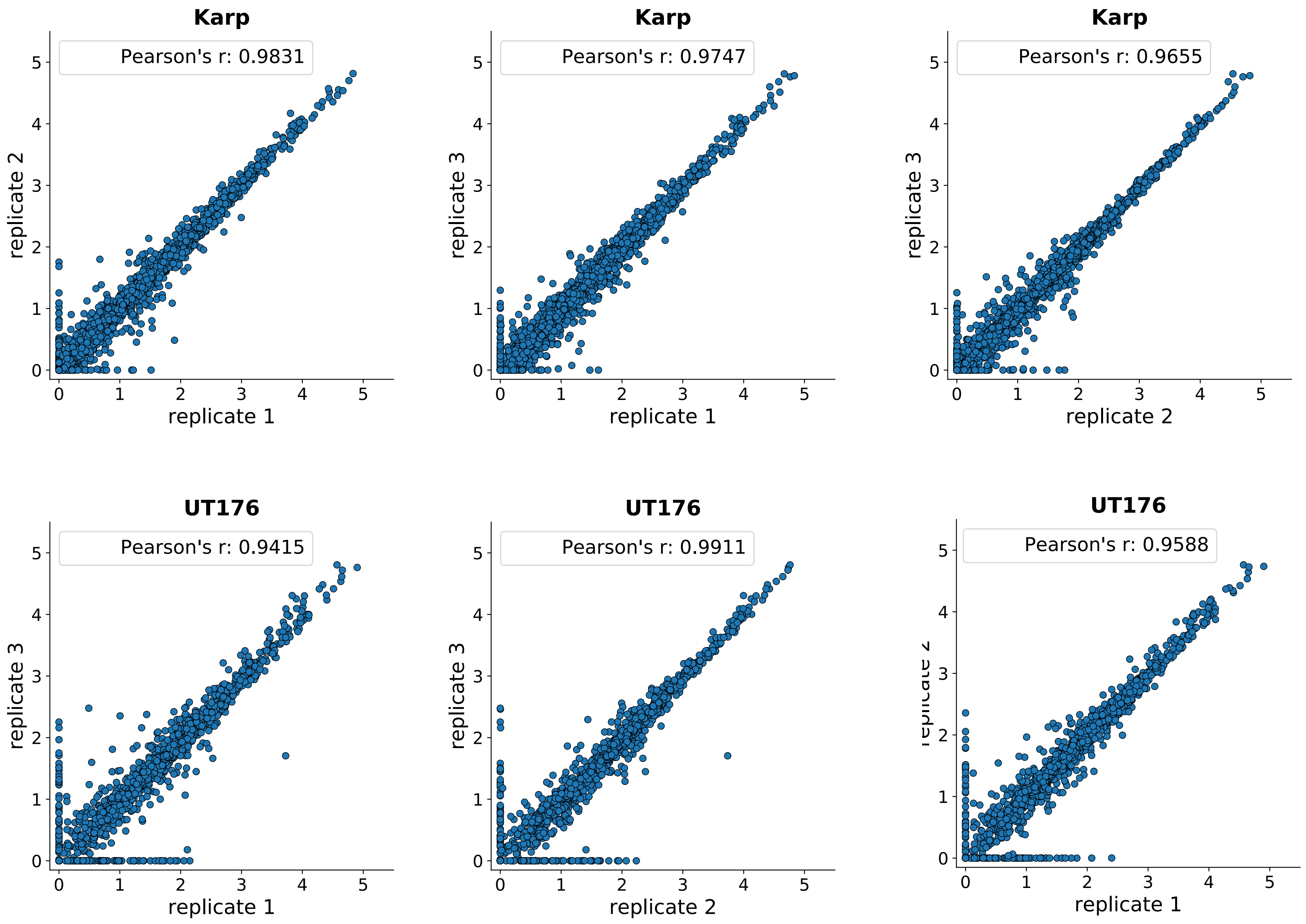

Supplementary Figure 4. Summary of data reproducibility. Comparison of replicate log10(TPM) values. Pearson correlation coefficient was calculated for untransformed TPM values.

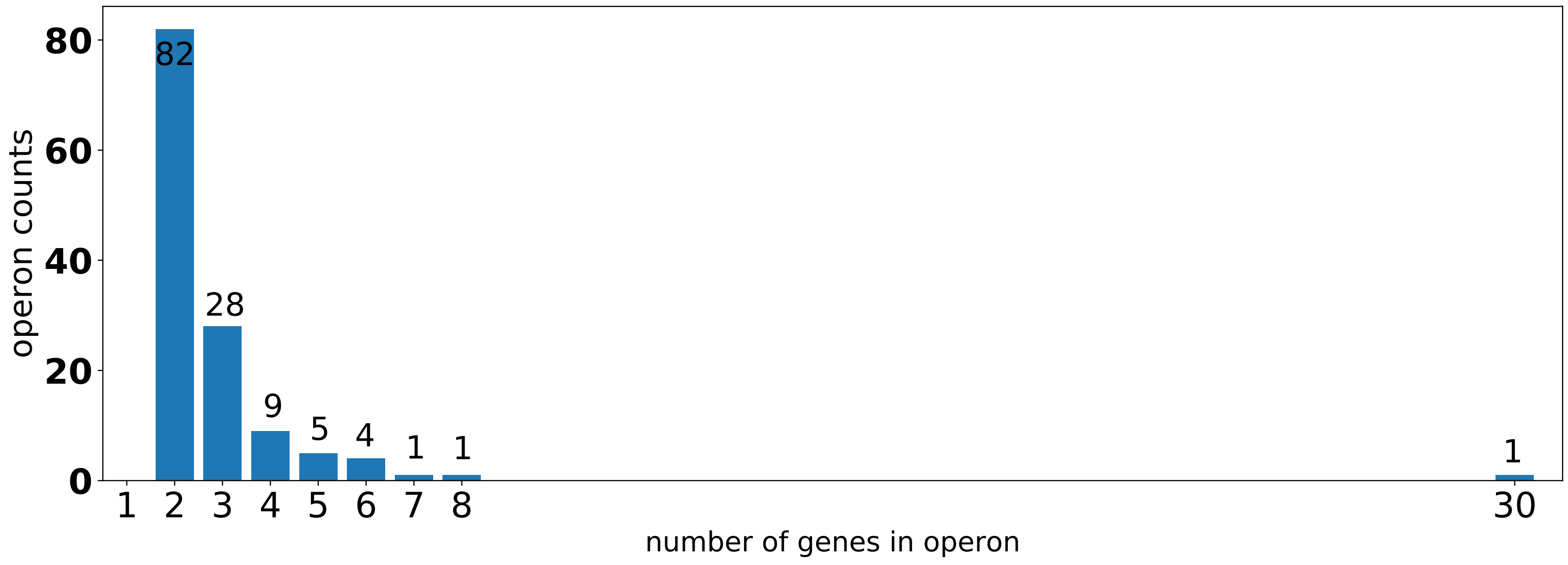

Supplementary Figure 5. Lengths of conserved operons in Ot

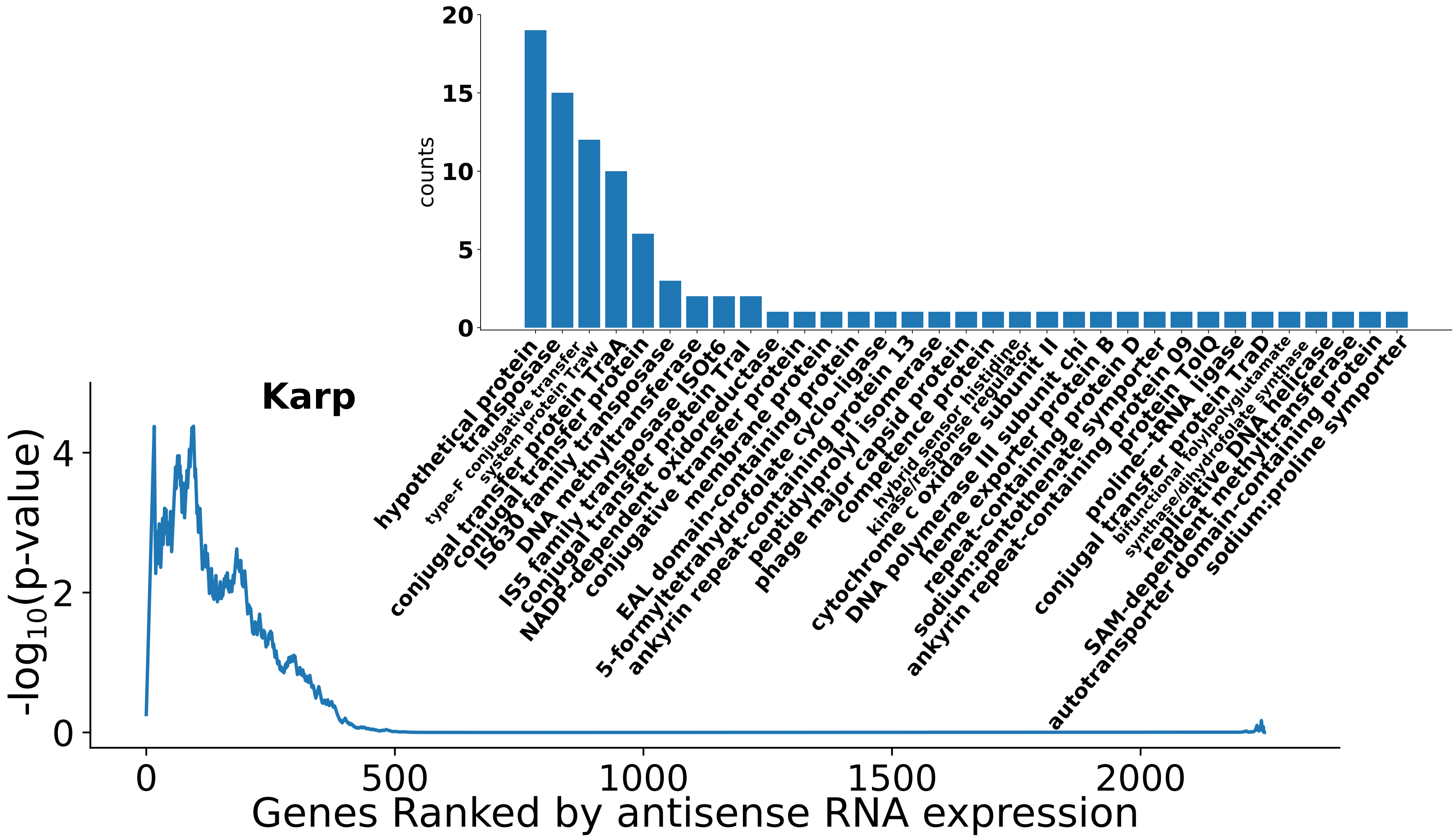

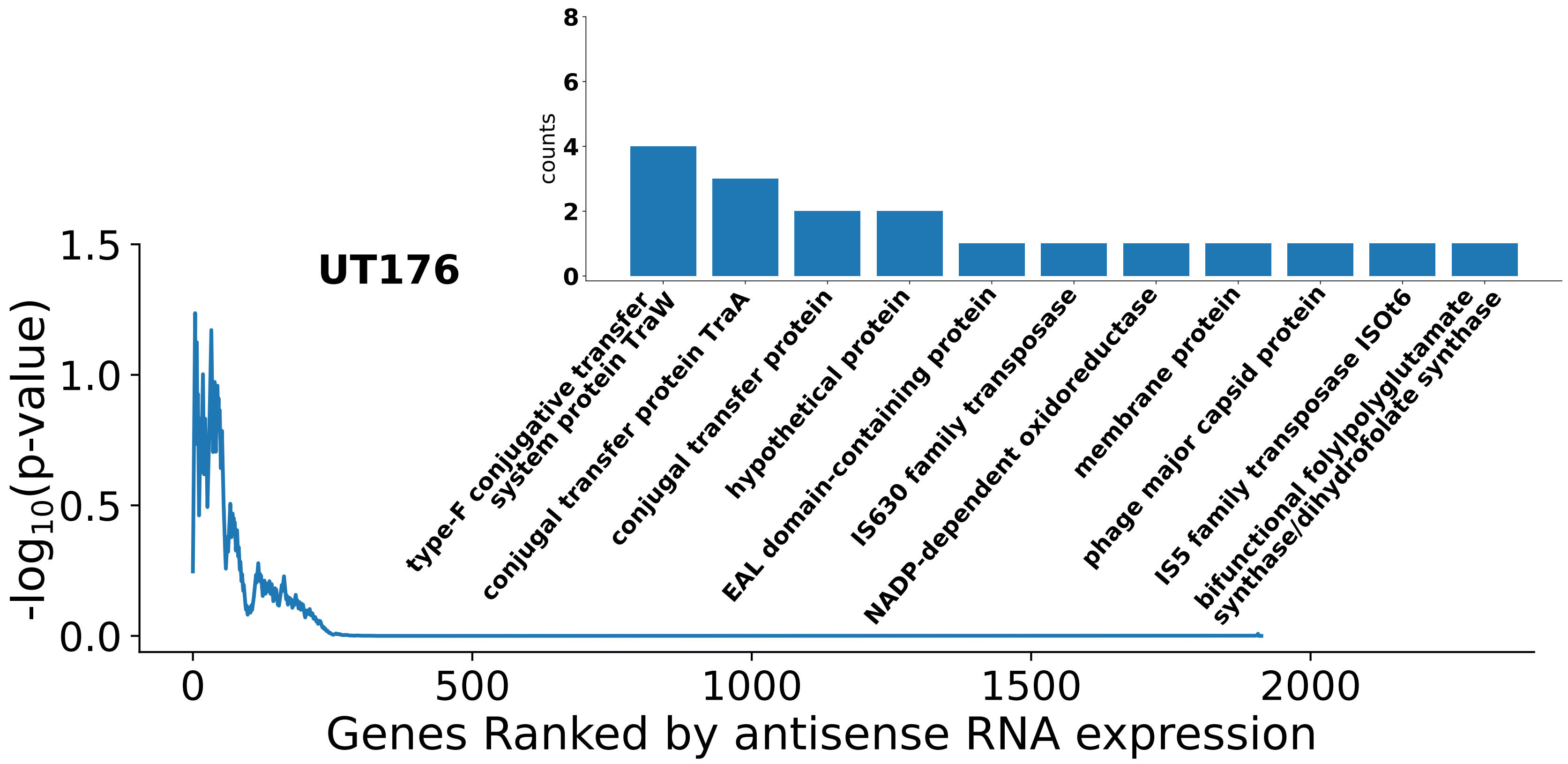

Supplementary Figure 6. Enrichment of RAGE genes in antisense expression. Genes were ranked by antisense read counts from highest to lowest, and a one-sided hypergeometric p-value was calculated at each position for enrichment of RAGE-annotated genes. Inset plots shows representative annotations for genes with high levels of antisense expression.

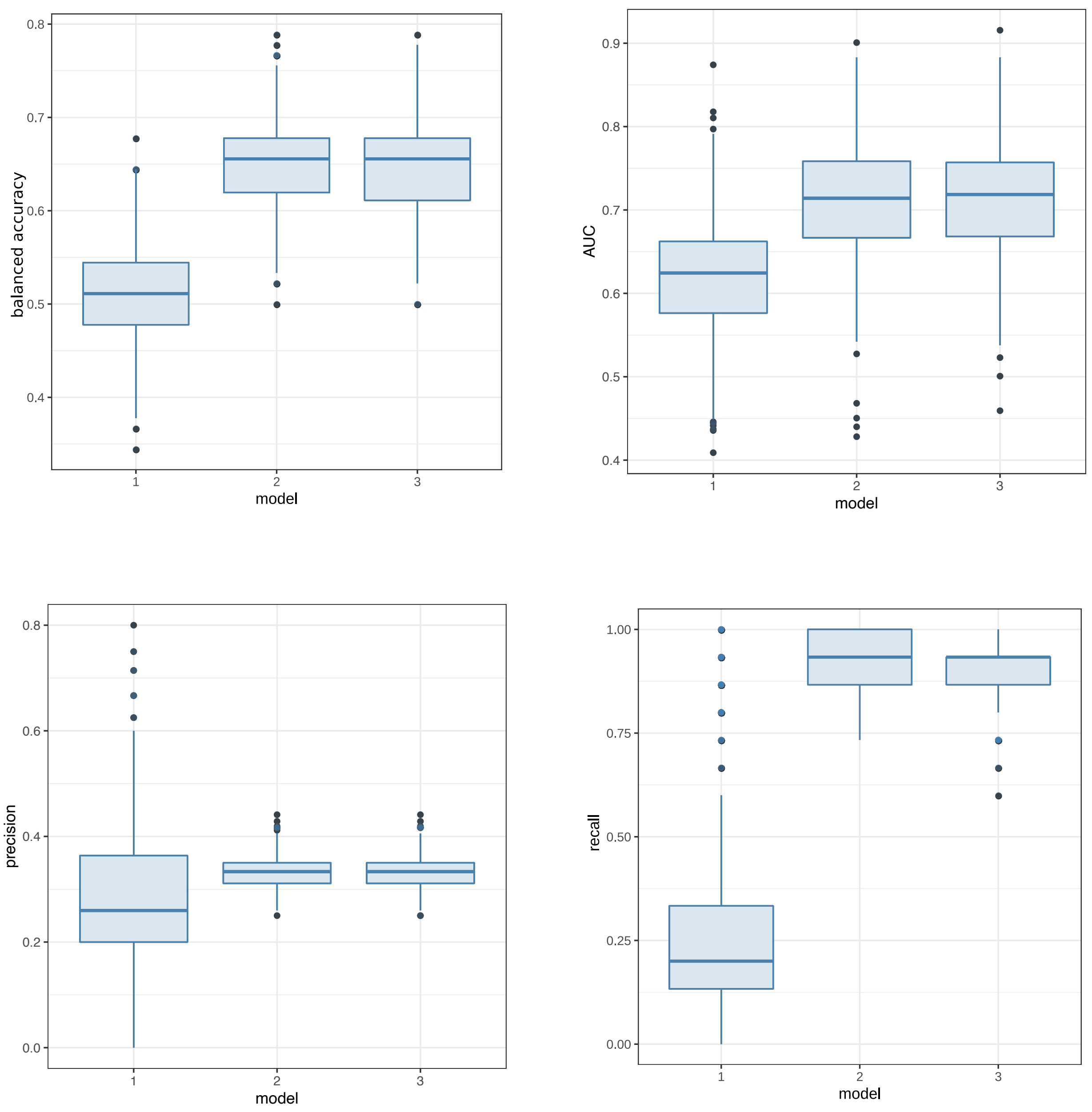

Supplementary Figure 7. Performance measures of logistic regression models in 500-fold cross validation. Models incorporating the antisense-sense read ratio (i.e. 2 and 3, see methods) perform better than model 1 based solely on sense expression. Box plot elements represent the following: center line, median; box limits, first and third quartiles; whiskers, furthest point within the 1.5x interquartile range; points, outliers. The lower and upper hinges in the boxplots correspond to the first and third quartiles of the data distribution, while the central line shows the median. The upper whisker extends from the hinge to the largest value no further than 1.5 * the inter-quartile range (IQR) from the hinge. The lower whisker extends from the hinge to the smallest value at most 1.5 * IQR of the hinge. Data points beyond 1.5 * IQR are plotted individually.

A.

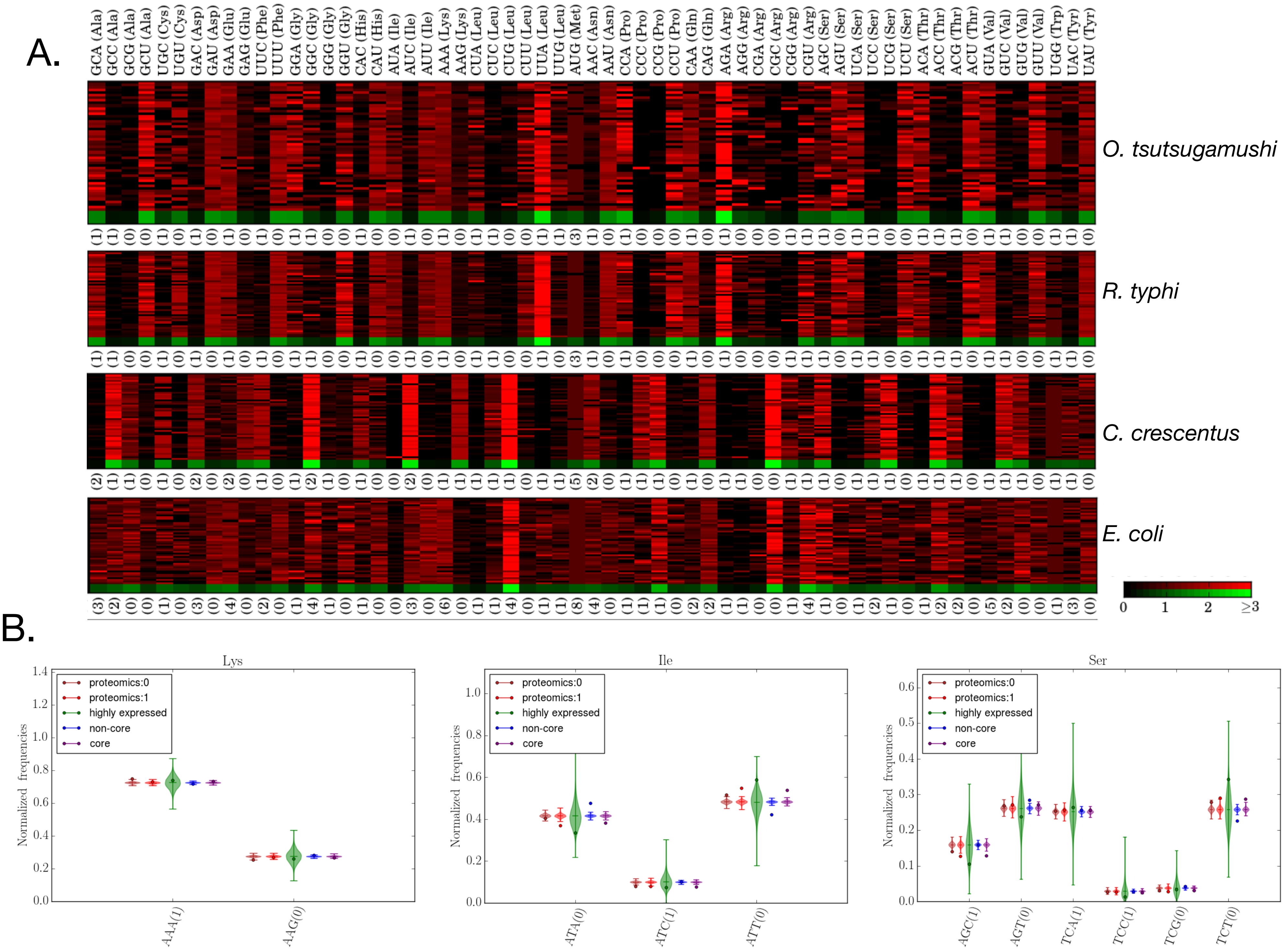

C.

amino acid	A	C	D	E	F	G	H	I	K	L	M	N	P	Q	R	S	T	V	W	Y
number of codons	4	2	2	2	2	4	2	3	2	6	1	2	4	2	6	6	4	4	1	2
bias between groups	Y	Y	N	N	N	Y	N	Y	N	N	-	N	Y	N	Y	Y	Y	Y	-	N
direction of genome-wide bias	S	N	N	P	N	S	N	N	P	S	-	N	S	P	S	S	S	S	-	N

Supplementary Figure 8. Codon bias in Ot. A. Genome-wide codon biases in *O. tsutsugamushi, R. typhi, C. crescentus* and *E. coli.* Genome-wide RSCU values are indicated by the green boxes, while the red boxes are the values for 50 randomly chosen individual genes. tRNA gene copy numbers are given in parentheses. B. Relative use of different codons by subsets of genes in Ot (Karp) that are absent in proteomics, detected in proteomics, highly expressed in proteomics (RpoB1, RpoB2, GroEL, DnaK, GdhA, HtpG, TSA56), encoded by non-core gene set or encoded by core gene set. Three illustrative examples are shown (Lys, Ile, Ser) with the full set of 20 amino sets given in Supp. Fig. 9. The relative codon frequency for each data set is shown as a point. The violin plots represent the distribution of the corresponding codon frequency, constructed by drawing 10,000 random sets of genes, each random set being equal in size to the corresponding data set. These distributions represent the relative codon frequencies due to random chance, and therefore the points that lie outside of these distributions can be considered statistically significant. C. Overview of relative use of codons to encode each amino acid in the different proteomics gene sets. The bias between groups (yes in green, no in red) indicates whether the different groups use the different possible codons differently. This is defined by a consistent trend between the not detected, detected and highly expressed groups. As an example, Lys in Supp. Fig. 8B=no, whilst Ile and Ser=yes. The direction of genome-wide bias indicates whether the genome-wide relative use of the different codon correlates with the presence or absence of cognate tRNA, or not. (positive correlation in green, negative correlation in red, split correlation in yellow). As an example Lys=positive, Ile=negative, Ser=split.

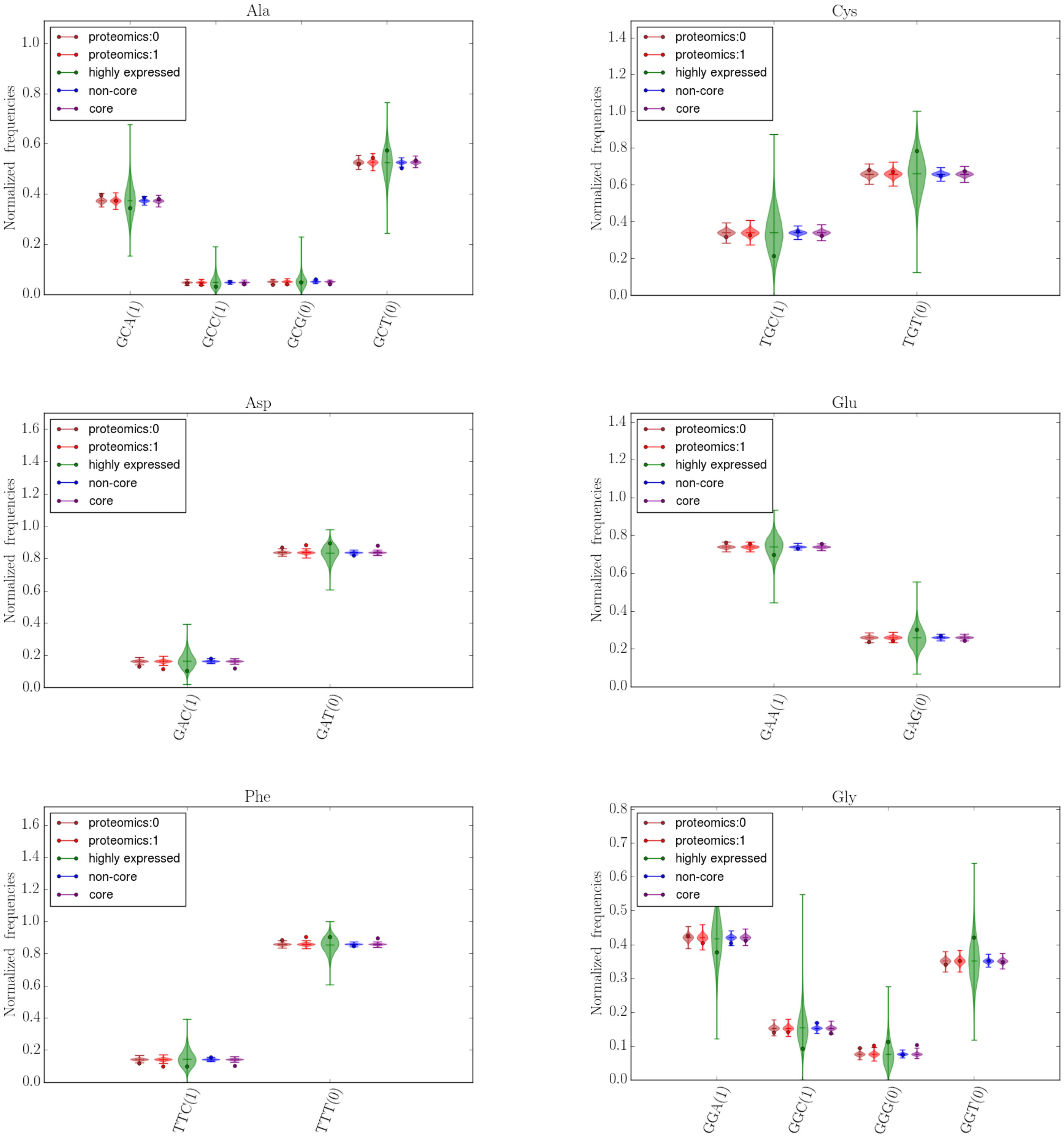

Supplementary Figure 9. Codon biases between different groups of OT_Karp genes. Relative use of different codons by subsets of genes in Ot (Karp) that are absent in proteomics, detected in proteomics, highly expressed in proteomics (RpoB1, RpoB2, GroEL, DnaK, GdhA, HtpG, TSA56), encoded by non-core gene set or encoded by core gene set. The relative codon frequency for each data set is shown as a point. The violin plots represent the distribution of the corresponding codon frequency, constructed by drawing 10,000 random sets of genes, each random set being equal in size to the corresponding data set. These distributions represent the relative codon frequencies due to random chance, and therefore the points that lie outside of these distributions can be considered statistically significant.

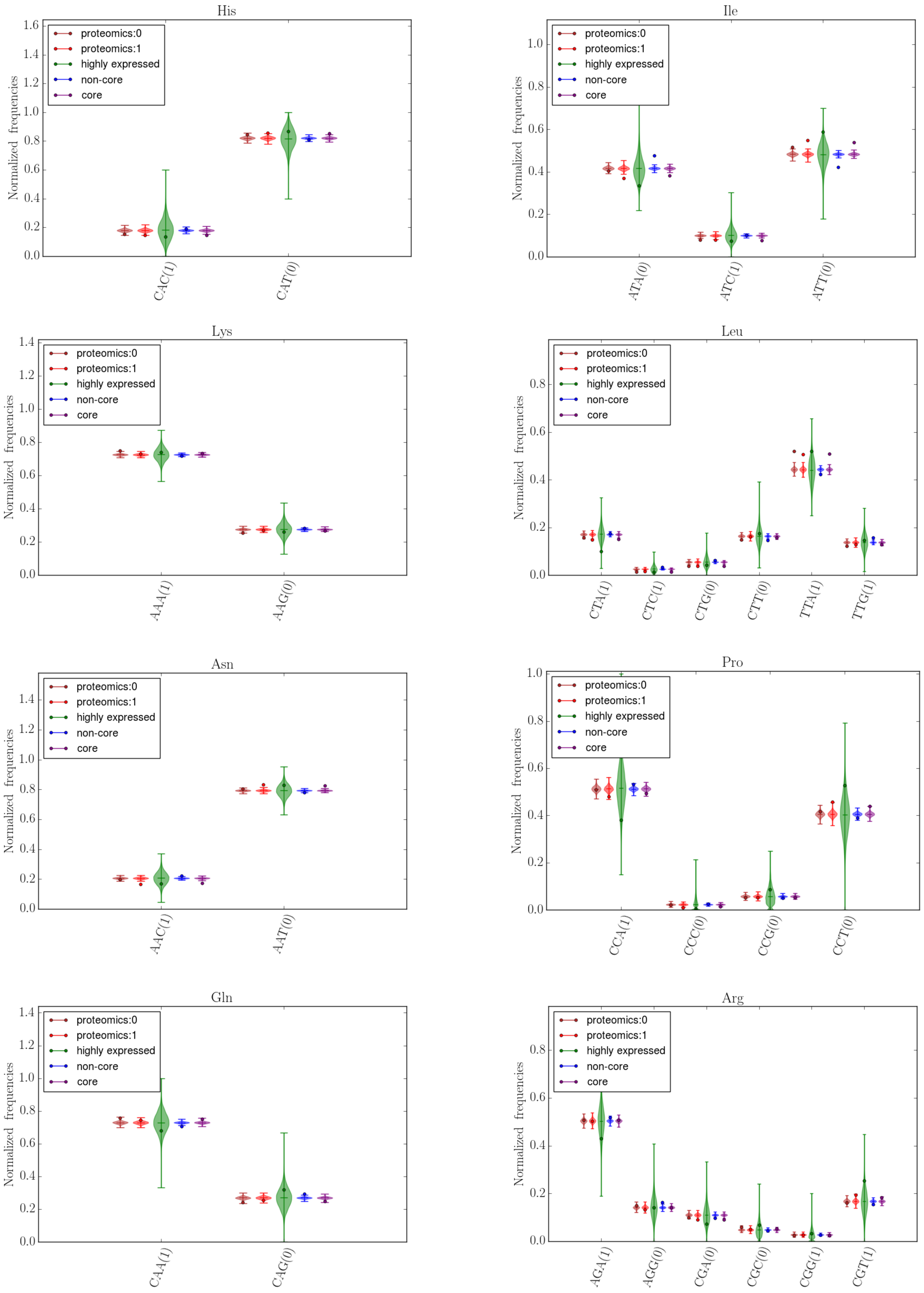

Supp. Fig. 9. continued

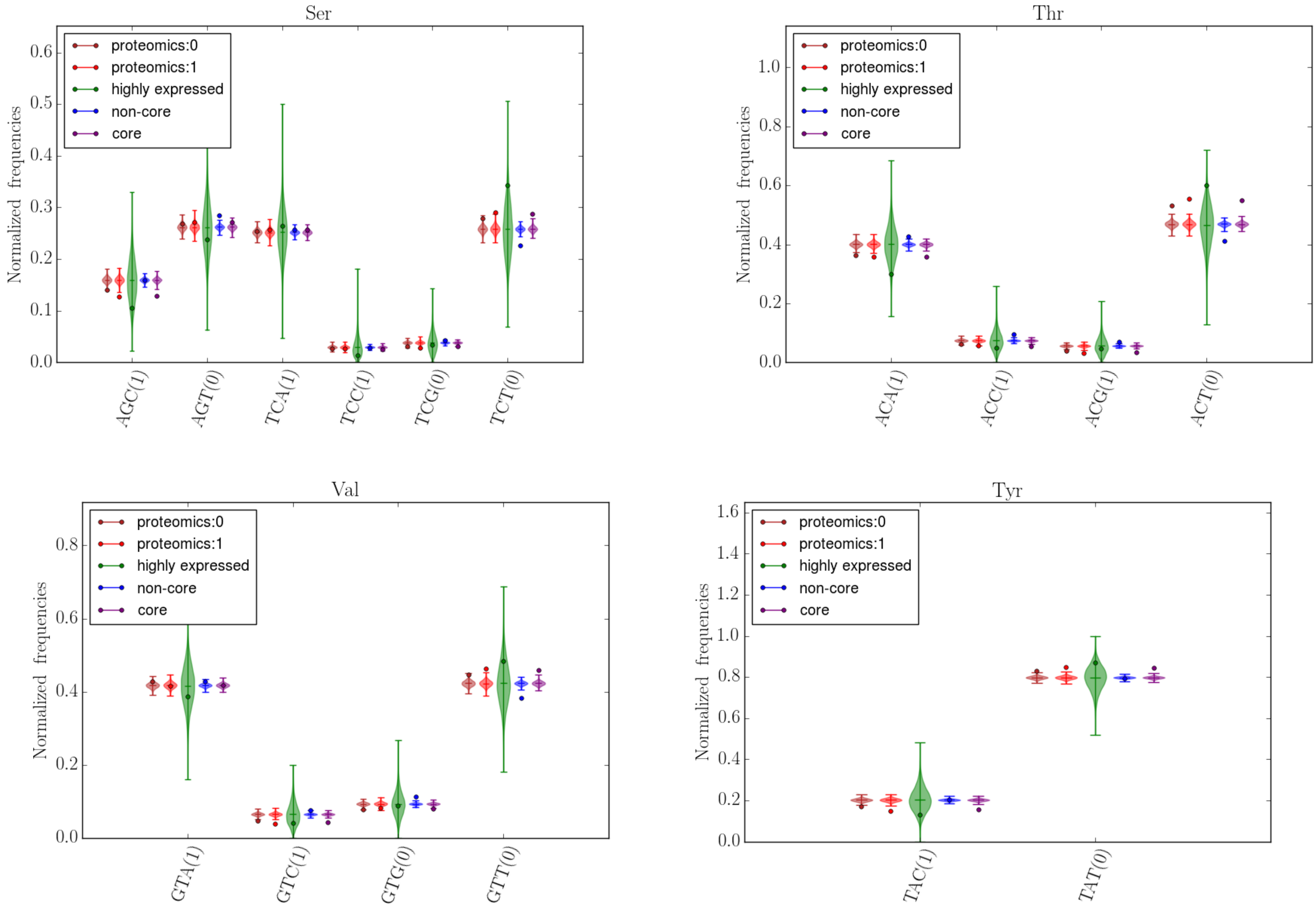

Supp. Fig. 9. continued

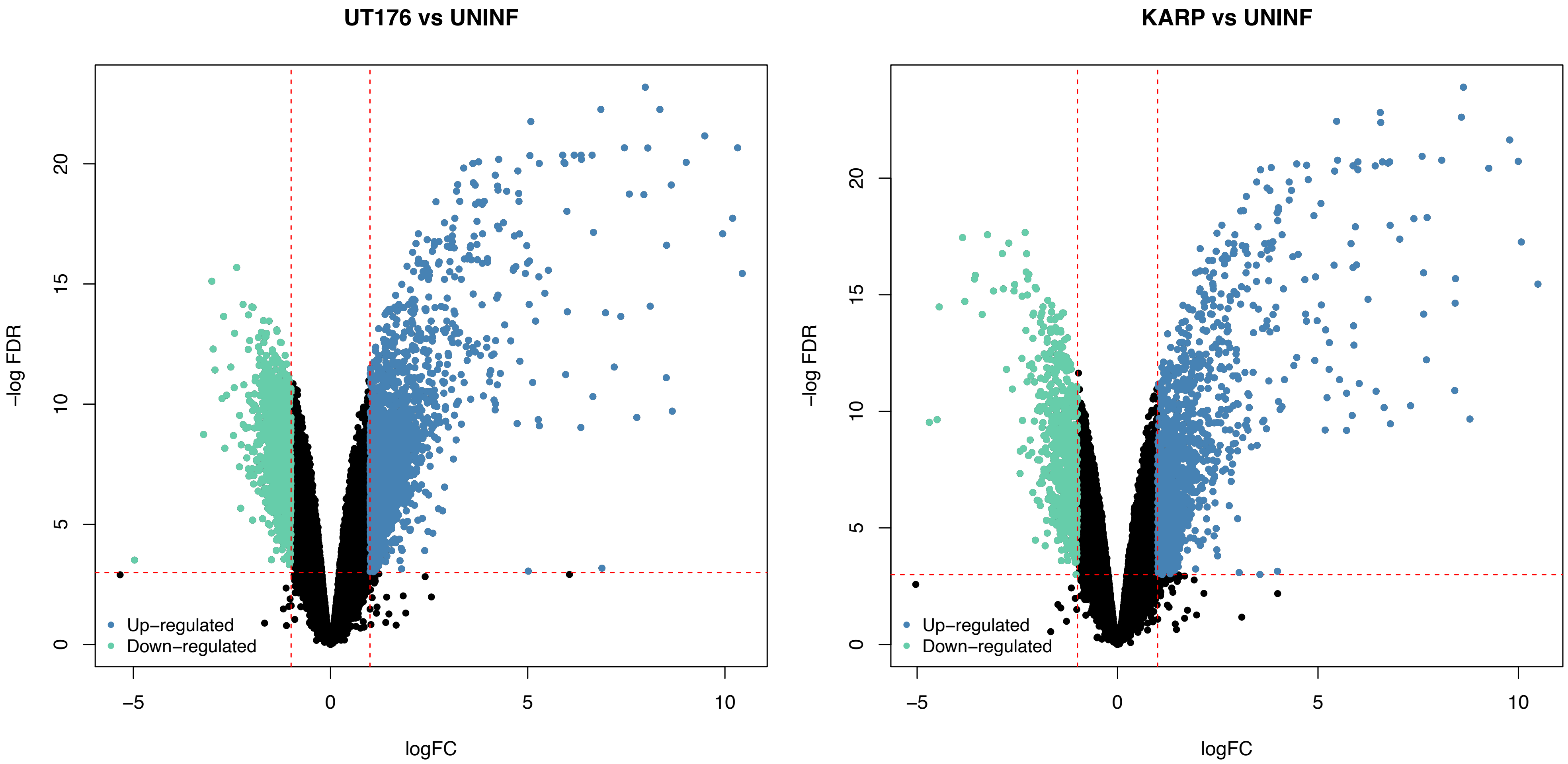

Supplementary Figure 10. Volcano plots showing differential expression of host genes in HUVEC cells infected with Ot_Karp and Ot_UT176. FDR-corrected two-sided p-values were calculated using the quasi-likelihood F-test in the edgeR R package.

Symbol	KARP	UT178
CXCL10	8.4	10.2
IFNB1	8.8	8.7
CXCL6	0.0	5.3
CCL5	4.1	4.8
CXCL11	4.4	4.7
CXCL5	0.0	4.2
CX3CL1	0.9	3.6
CCL20	-1.2	3.6
IL7R	3.8	3.5
TNFSF10	3.6	3.4
CXCL1	0.0	3.2
IL1RL1	0.5	3.0
CXCL8	-2.0	2.9
CXCL3	0.0	2.9
IL18R1	0.5	2.7
TNFAIP3	1.2	2.7
IL34	0.0	2.6
IL6	0.0	2.5
IL3RA	2.1	2.2
IL15RA	2.1	2.1
IL1A	0.0	2.1
TNFSF13B	1.7	2.1
IL13RA2	5.9	2.0
CXCL2	-0.5	1.9
IL15	1.1	1.7
TNFRSF9	0.0	1.7
IL1R1	0.0	1.7
TNFAIP2	0.6	1.6
IL33	6.6	1.5
TNFSF18	0.0	1.4
IL12A	0.0	1.3
TNFRSF1B	0.9	1.3
IL18BP	1.3	1.3
CCL2	-1.5	1.1
TNFRSF25	1.2	1.0
IL11RA	0.7	0.9

Symbol	KARP	UT178
TNFAIP6	0.0	0.9
TNFAIP8	0.0	0.9
IFNAR2	0.8	0.8
IL20RB	0.0	0.8
CXCL16	1.1	0.8
TNFRSF11A	1.4	0.8
IL6R	1.1	0.8
TNFRSF14	0.0	0.7
IL4R	0.0	0.6
IL4I1	0.0	0.6
IFNGR2	-0.3	0.6
TNFAIP8L1	0.0	0.5
CXCR4	0.0	0.5
IL1RAP	0.0	0.5
IL1RAPL2	1.8	0.0
TNFAIP8L3	1.3	0.0
IL1RAPL1	1.0	0.0
TNFRSF10A	0.8	0.0
TNFRSF6B	0.7	0.0
IL6ST	-0.4	0.0
TNFRSF12A	-0.4	0.0
IL17RA	-0.4	0.0
TNFRSF10B	-0.5	0.0
IL6STP1	-0.6	0.0
IL17D	-0.6	0.0
TNFRSF19	-0.8	0.0
TNFSF15	-2.6	0.0
TNFRSF1A	0.4	-0.3
TNFRSF10C	-0.4	-0.4
TNFSF12	0.0	-0.5
IL13RA1	-1.0	-0.5
IL32	-1.1	-0.7
TNFRSF21	0.3	-0.7
TNFSF4	0.9	-1.4
TNFRSF10D	-1.6	-1.7

Supplementary Figure 11. Differential cytokine and chemokine gene expression pattern in Ot_KARP or Ot_UT176 infected HUVEC cells measured by RNAseq.

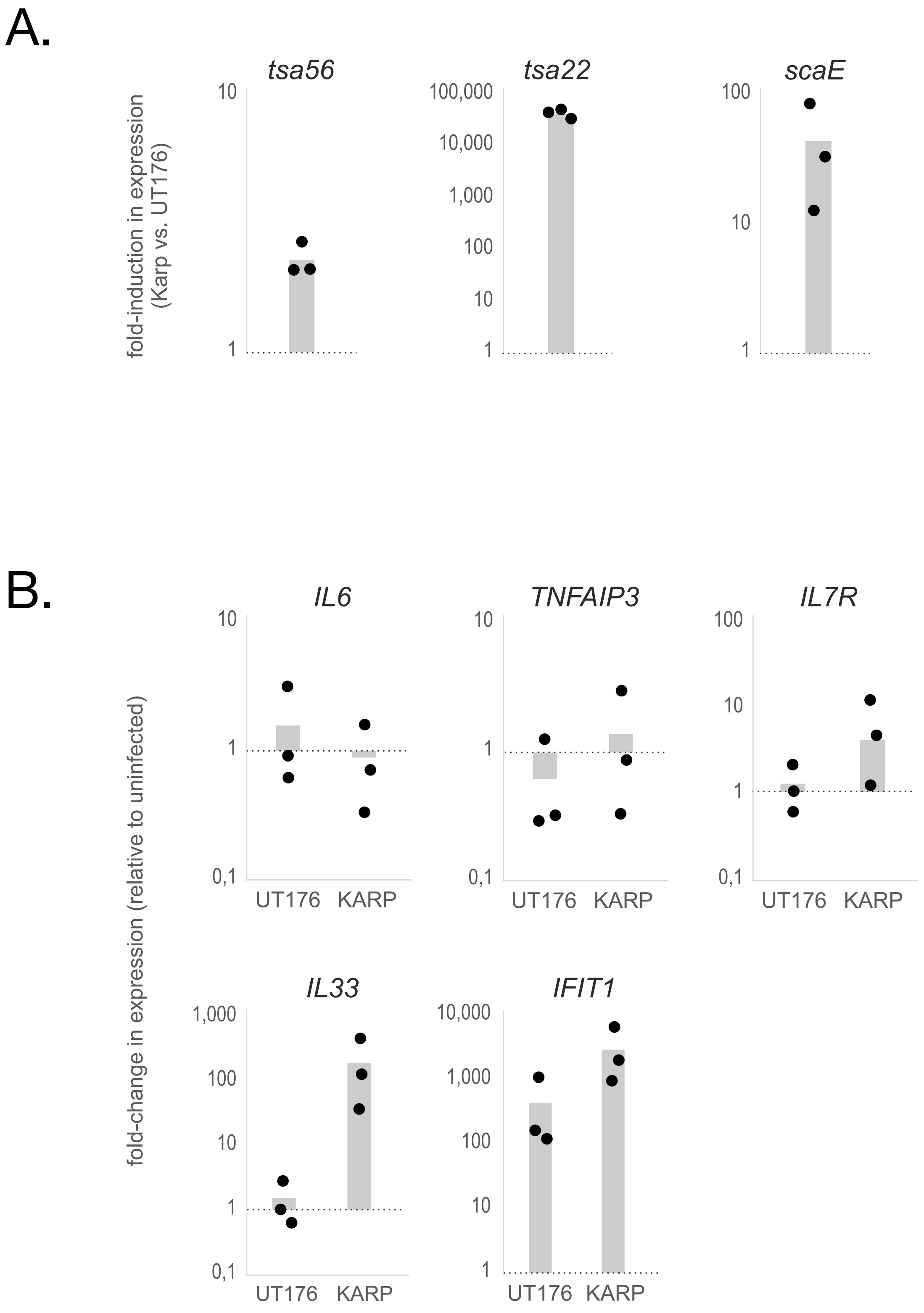

Supplementary Figure 12. Differential analysis of host and bacterial genes. A. qRT-PCR of selected bacterial genes in HUVEC cells infected with Ot_Karp or Ot_UT176. The expression level was normalised using 7S mRNA. B. qRT-PCR of host genes in HUVEC cells infected with Ot_Karp or Ot_UT176. The individual values and mean of three biologically independent replicates are shown.

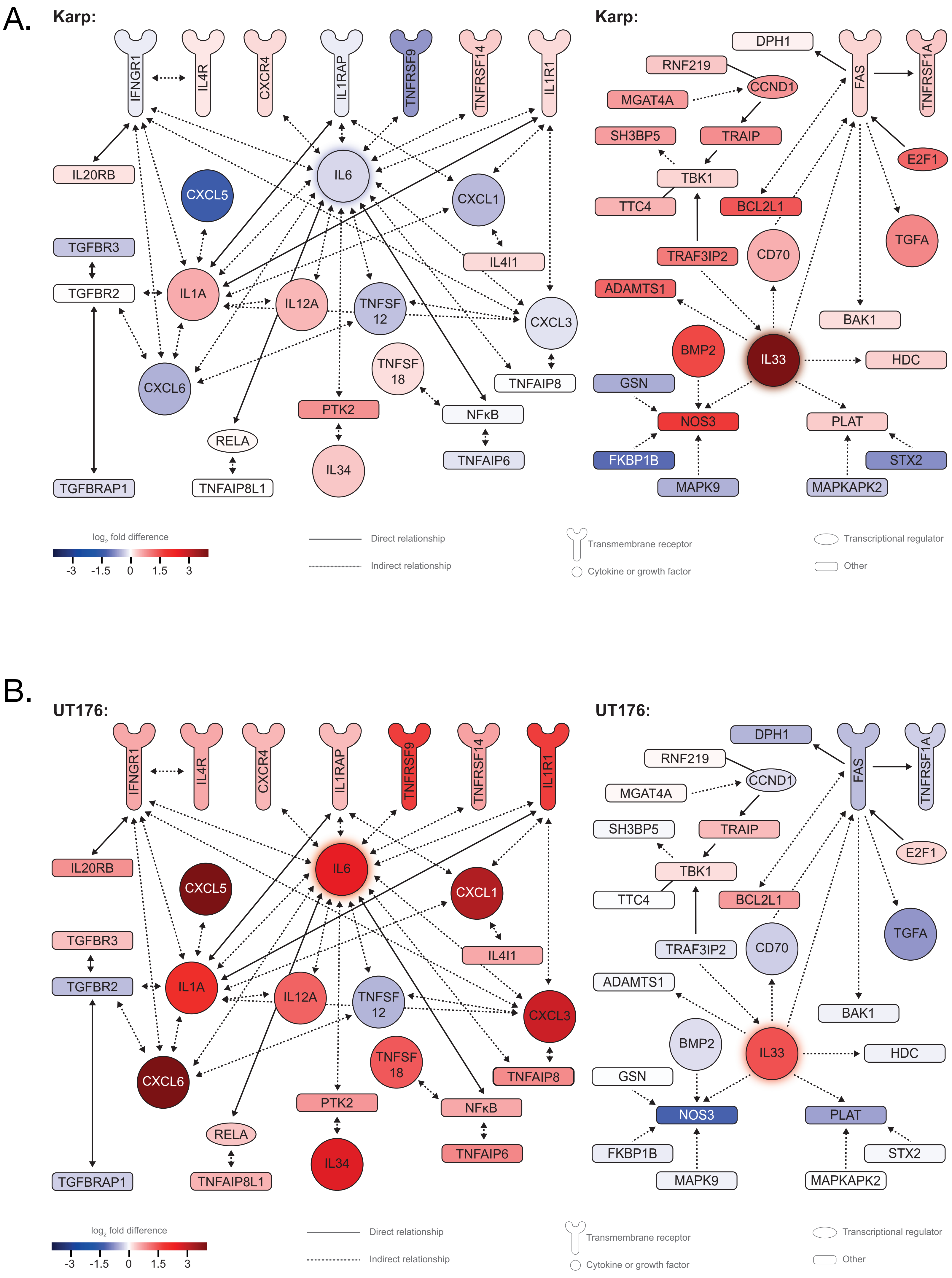

Supplementary Figure 13. Karp and UT176 lead to up-regulation of distinct networks in HUVEC cells.

A.

Symbol	Entrez Gene Name	Expr Log Ratio (UT176 vs	Expr Log Ratio (KARP vs	Location	Gene ID-Huma	Gene ID-Mous
Cot	mitogen-activated protein kinase kinase kinase 8	1.70782	0.144809	Cytoplasm	1326	26410
RelB	RELB proto-oncogene, NF-kB subunit	1.28783	-0.03004	Nucleus	5971	19698
MEKK1	mitogen-activated protein kinase kinase kinase 1	0.92341	-0.0359	Cytoplasm	4214	26401
JNK1	mitogen-activated protein kinase 8	0.817456	0.423134	Cytoplasm	5599	26419
NF-ϰB2 p100	nuclear factor kappa B subunit 2	0.766144	-0.39373	Nucleus	4791	18034
NF-ϰB1	nuclear factor kappa B subunit 1	0.599586	-0.03203	Nucleus	4790	18033
β-TrCP	beta-transducin repeat containing E3 ubiquitin protein ligase	0.465374	0.146903	Cytoplasm	8945	12234
MALT1	MALT1 paracaspase	0.404342	0.115903	Cytoplasm	10892	240354
p65/RelA	RELA proto-oncogene, NF-kB subunit	0.400752	0.054767	Nucleus	5970	19697
LTBR	lymphotoxin beta receptor	-0.3528	-0.22677	Plasma Membrane	4055	17000
IKKα	conserved helix-loop-helix ubiquitous kinase	-0.39103	0.0463	Cytoplasm	1147	12675
Bcl10	B cell CLL/lymphoma 10	-0.67364	-0.04115	Cytoplasm	8915	12042

Expression of NFkB pathway genes

B.

Sym bol	Entrez Gene Name	Expr Log Ratio (UT176 vs UnInf)	Expr Log Ratio (KARP vs UnInf)	Locatio n	Gene ID-Human	Gene ID-Mouse
PPA Rα	peroxisome proliferator activated receptor alpha	0.955636	0.256633	Nucleus	5465	19013
SIR Pα	signal regulatory protein alpha	0.855516	0.231361	Plasma Membra ne	140885	19261
ME K1	mitogen-activated protein kinase kinase 1	0.726498	0.240664	Cytoplas m	5604	26395
CBP	CREB binding protein	0.365126	0.187724	Nucleus	1387	12914

Expression of genes involved in NOS2 production

Supp Fig 14. Differential regulation of inflammatory pathways by UT176 and Karp. A. shows expression of NFkB pathway genes in UT176- and Karp-infected host cells. B. shows expression of host genes associated with NOS2 production. Red indicates increased expression relative to uninfected cells, blue indicates decreased expression.

A. Expression of host genes associated with differentiation of mononuclear leukocytes

ID	Genes in dataset	Expr Log Ratio (UT176 vs Uninf)	Expr Log Ratio (KARP vs Uninf)	Prediction in UT176 (based on measurement direction)
ENSG00000171855.6	*IFNB1*	8.664	8.790328	Increased
ENSG00000164400.5	*CSF2*	7.764	-1.667	Increased
ENSG00000184979.9	*USP18*	5.078	5.464608	Increased
ENSG00000108342.12	*CSF3*	5.037	2.804455	Increased
ENSG00000114251.13	*WNT5A*	4.976	3.61382	Increased
ENSG00000271503.5	*CCL5*	4.801	4.102429	Increased
ENSG00000137462.6	*TLR2*	4.416	-0.21786	Increased
ENSG00000163735.6	*CXCL5*	4.174	-1.41573	Increased
ENSG00000057657.14	*PRDM1*	3.961	1.080978	Increased
ENSG00000128917.6	*DLL4*	3.512	1.850144	Increased
ENSG00000168685.14	*IL7R*	3.5	3.802424	Increased
ENSG00000121858.10	*TNFSF10*	3.376	3.567363	Increased
ENSG00000163739.4	*CXCL1*	3.236	-0.44897	Increased
ENSG00000137752.22	*CASP1*	3.15	2.684897	Increased
ENSG00000130775.15	*THEMIS2*	3.135	3.231943	Increased
ENSG00000185507.19	*IRF7*	3.022	3.875153	Increased
ENSG00000169429.10	*CXCL8*	2.907	-1.96917	Increased
ENSG00000028277.20	*POU2F2*	2.764	0.453238	Increased
ENSG00000115604.10	*IL18R1*	2.736	0.499293	Increased
ENSG00000138378.17	*STAT4*	2.695	2.145555	Increased
ENSG00000157368.10	*IL34*	2.595	0.369739	Increased
ENSG00000170298.15	*LGALS9B*	2.528	2.965212	Increased
ENSG00000136244.11	*IL6*	2.503	-0.23512	Increased
ENSG00000134363.11	*FST*	2.481	1.895928	Increased
ENSG00000109906.13	*ZBTB16*	2.408	0.917825	Increased
ENSG00000089041.16	*P2RX7*	2.275	0.379212	Increased
ENSG00000204632.11	*HLA-G*	2.231	2.459558	Increased
ENSG00000184371.13	*CSF1*	2.22	1.125217	Increased
ENSG00000134470.19	*IL15RA*	2.14	2.119752	Increased
ENSG00000115415.18	*STAT1*	2.106	2.370024	Increased
ENSG00000115008.5	*IL1A*	2.073	0.577578	Increased
ENSG00000102524.11	*TNFSF13B*	2.069	1.73795	Increased
ENSG00000164330.16	*EBF1*	2.065	2.951442	Increased
ENSG00000123496.7	*IL13RA2*	2.024	5.897182	Increased
ENSG00000125347.13	*IRF1*	2.022	1.618451	Increased
ENSG00000134321.11	*RSAD2*	10.321	9.995906	Affected
ENSG00000169245.5	*CXCL10*	10.195	8.430441	Affected
ENSG00000107201.9	*DDX58*	4.771	3.772748	Affected
ENSG00000164342.12	*TLR3*	4.216	2.7201	Affected
ENSG00000168961.16	*LGALS9*	3.756	4.338328	Affected
ENSG00000152689.17	*RASGRP3*	3.317	2.743657	Affected
ENSG00000048052.21	*HDAC9*	2.949	1.379593	Affected
ENSG00000170581.13	*STAT2*	2.748	2.681712	Affected
ENSG00000134215.15	*VAV3*	2.603	2.987545	Affected
ENSG00000169248.12	*CXCL11*	4.66	4.371531	Decreased
ENSG00000240065.7	*PSMB9*	3.567	3.780511	Decreased
ENSG00000166923.10	*GREM1*	3.214	-2.05055	Decreased
ENSG00000115602.16	*IL1RL1*	2.959	0.520912	Decreased
ENSG00000118503.14	*TNFAIP3*	2.674	1.151952	Decreased
ENSG00000140464.19	*PML*	2.167	1.947424	Decreased

B. Expression of host genes associated with leukocyte proliferation

ID	Genes in dataset	Expr Log Ratio (UT176 vs UnInf)	Expr Log Ratio (KARP vs UnInf)	Prediction in UT176 (based on measurement direction)
ENSG00000171855.6	*IFNB1*	8.664	8.790328	Increased
ENSG00000164400.5	*CSF2*	7.764	-1.667	Increased
ENSG00000187608.8	*ISG15*	5.293	5.416531	Increased
ENSG00000108342.12	*CSF3*	5.037	2.804455	Increased
ENSG00000271503.5	*CCL5*	4.801	4.102429	Increased
ENSG00000137462.6	*TLR2*	4.416	-0.21786	Increased
ENSG00000172183.14	*ISG20*	4.384	4.897459	Increased
ENSG00000164342.12	*TLR3*	4.216	2.7201	Increased
ENSG00000168685.14	*IL7R*	3.5	3.802424	Increased
ENSG00000172348.14	*RCAN2*	2.909	1.89052	Increased
ENSG00000100234.11	*TIMP3*	2.788	1.065608	Increased
ENSG00000028277.20	*POU2F2*	2.764	0.453238	Increased
ENSG00000138378.17	*STAT4*	2.695	2.145555	Increased
ENSG00000134215.15	*VAV3*	2.603	2.987545	Increased
ENSG00000157368.10	*IL34*	2.595	0.369739	Increased
ENSG00000170091.10	*NSG2*	2.557	3.988829	Increased
ENSG00000136244.11	*IL6*	2.503	-0.23512	Increased
ENSG00000026950.16	*BTN3A1*	2.475	2.699843	Increased
ENSG00000170166.5	*HOXD4*	2.286	2.00549	Increased
ENSG00000089041.16	*P2RX7*	2.275	0.379212	Increased
ENSG00000184371.13	*CSF1*	2.22	1.125217	Increased
ENSG00000185950.8	*IRS2*	2.142	-0.59384	Increased
ENSG00000134470.19	*IL15RA*	2.14	2.119752	Increased
ENSG00000113319.11	*RASGRF2*	2.099	1.634606	Increased
ENSG00000115008.5	*IL1A*	2.073	0.577578	Increased
ENSG00000102524.11	*TNFSF13B*	2.069	1.73795	Increased
ENSG00000114251.13	*WNT5A*	4.976	3.61382	Affected
ENSG00000162692.10	*VCAM1*	4.467	-0.03961	Affected
ENSG00000168961.16	*LGALS9*	3.756	4.338328	Affected
ENSG00000152689.17	*RASGRP3*	3.317	2.743657	Affected
ENSG00000177409.11	*SAMD9L*	3.277	2.927656	Affected
ENSG00000115602.16	*IL1RL1*	2.959	0.520912	Affected
ENSG00000023445.13	*BIRC3*	2.889	1.028695	Affected
ENSG00000163734.4	*CXCL3*	2.872	-0.17656	Affected
ENSG00000152217.16	*SETBP1*	2.574	1.333599	Affected
ENSG00000140464.19	*PML*	2.167	1.947424	Affected
ENSG00000169245.5	*CXCL10*	10.195	8.430441	Decreased
ENSG00000175899.14	*A2M*	5.199	4.163204	Decreased
ENSG00000131203.12	*IDO1*	4.034	2.870018	Decreased
ENSG00000057657.14	*PRDM1*	3.961	1.080978	Decreased
ENSG00000173193.13	*PARP14*	3.713	3.461054	Decreased
ENSG00000115009.11	*CCL20*	3.616	-1.19333	Decreased
ENSG00000128917.6	*DLL4*	3.512	1.850144	Decreased
ENSG00000121858.10	*TNFSF10*	3.376	8.790328	Decreased
ENSG00000169429.10	*CXCL8*	2.907	-1.667	Decreased
ENSG00000118503.14	*TNFAIP3*	2.674	5.416531	Decreased
ENSG00000170298.15	*LGALS9B*	2.528	2.804455	Decreased
ENSG00000109906.13	*ZBTB16*	2.408	4.102429	Decreased
ENSG00000204632.11	*HLA-G*	2.231	-0.21786	Decreased
ENSG00000115415.18	*STAT1*	2.106	4.897459	Decreased

Supplementary Figure 15. Differential activation of inflammatory pathways by UT176 and Karp. A. shows expression of host genes associated with differentiation of mononuclear leukocytes B. shows expression of host genes associated with leukocyte proliferation. The predicted effect on leukocyte differentiation/proliferation is shown, based on the gene expression in response to UT176 infection. Red indicates increased expression relative to uninfected cells, blue indicates decreased expression.

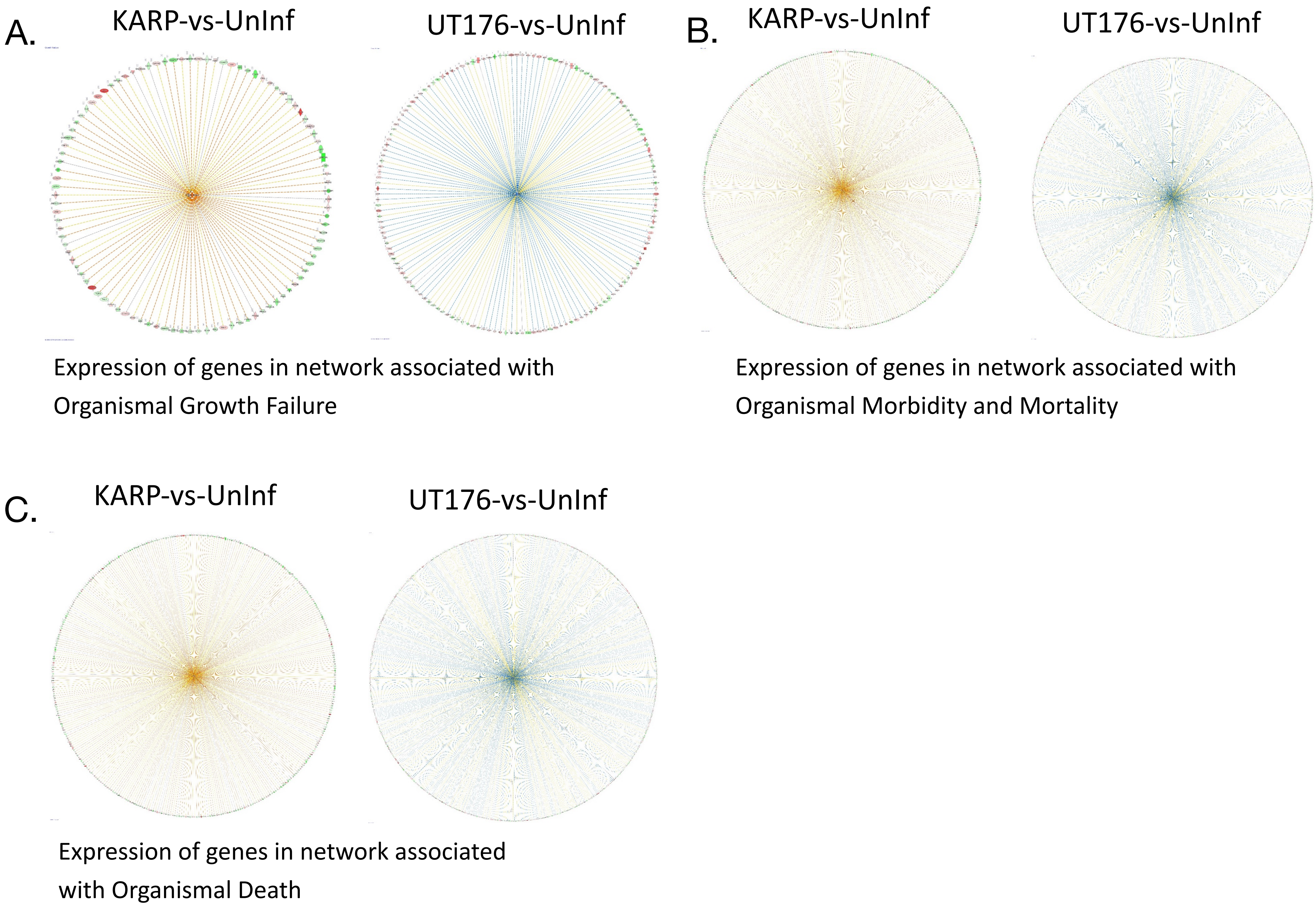

Supplementary Figure 16. Ot_Karp up-regulates networks associated with (A) organismal growth failure, (B) morbidity and mortality and (C) death. Connections coloured in red are up-regulated whilst connections in blue are down-regulated.

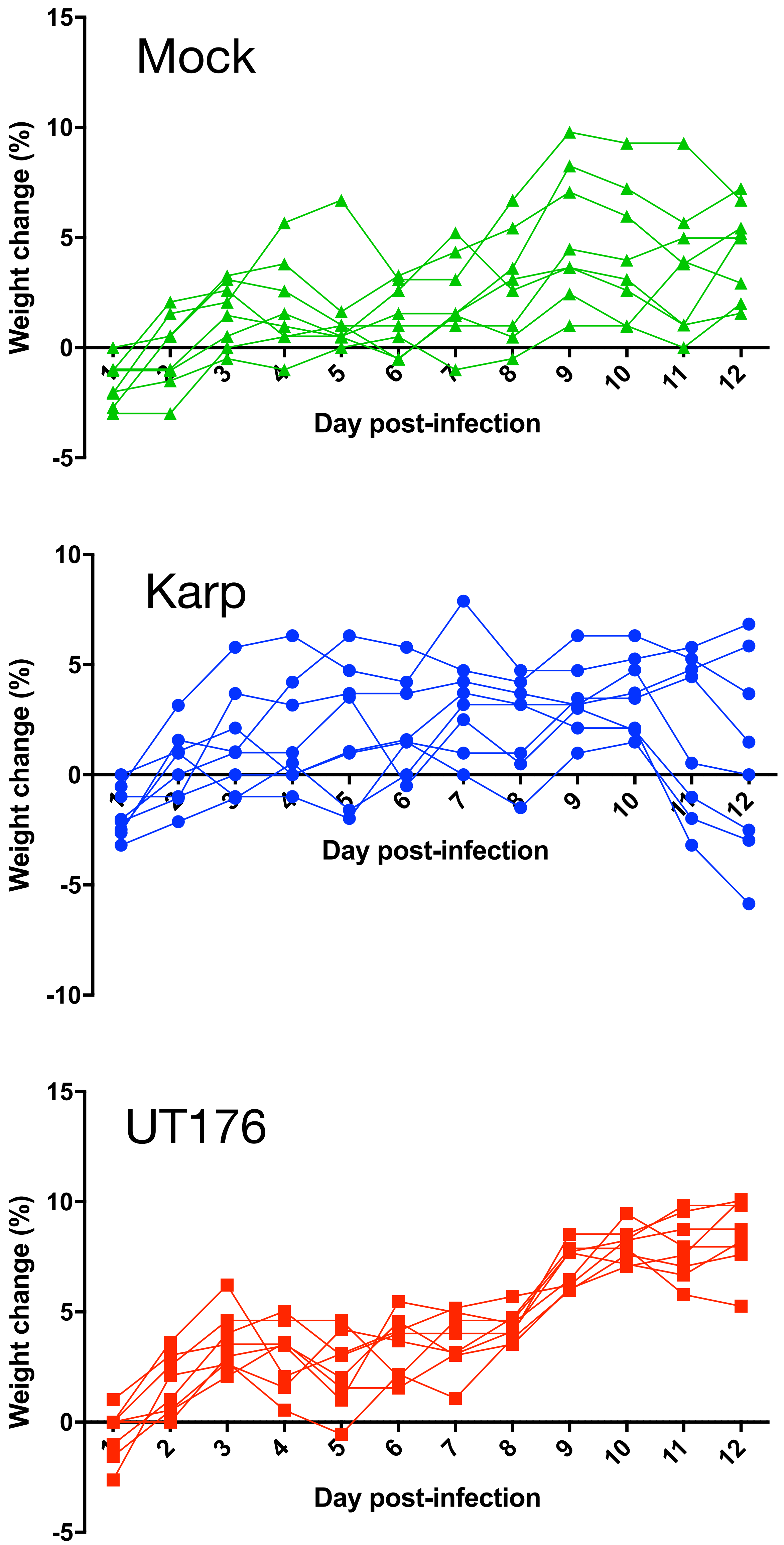

Supplementary Figure 17. Daily weight measurements of individual mice infected with Ot. Source data are provided as a Source Data file.

A. Mouse clinical observation scoring system

Parameter		CODE	SCORE
**Group Appetite**	ate all 6-12 chows	0	0
	ate 1-5 chows	1	1
	ate 0 to 1/2 chow	2	2
**Activity**	move around	0	0
	locomotion after slight stimulation	A1	1
	move slowly after moderate stimulation	A2	2
	unable to move	A3	3
**Hair coat**	well-groomed hair coat	0	0
	rough hair coat	R1	1
	ungroomed, very rough hair coat, and dirty	R2	2
**Total score**	7		

B.

Lesion scoring system

0 = Normal tissue
1 = Minimal lesion severity and extent
2 = Mild
3 = Moderate
4 = Marked
5 = Severe

Supplementary Figure 18. System used for scoring (A) clinical observations and (B) Lesions in H&E stained tissue sections.

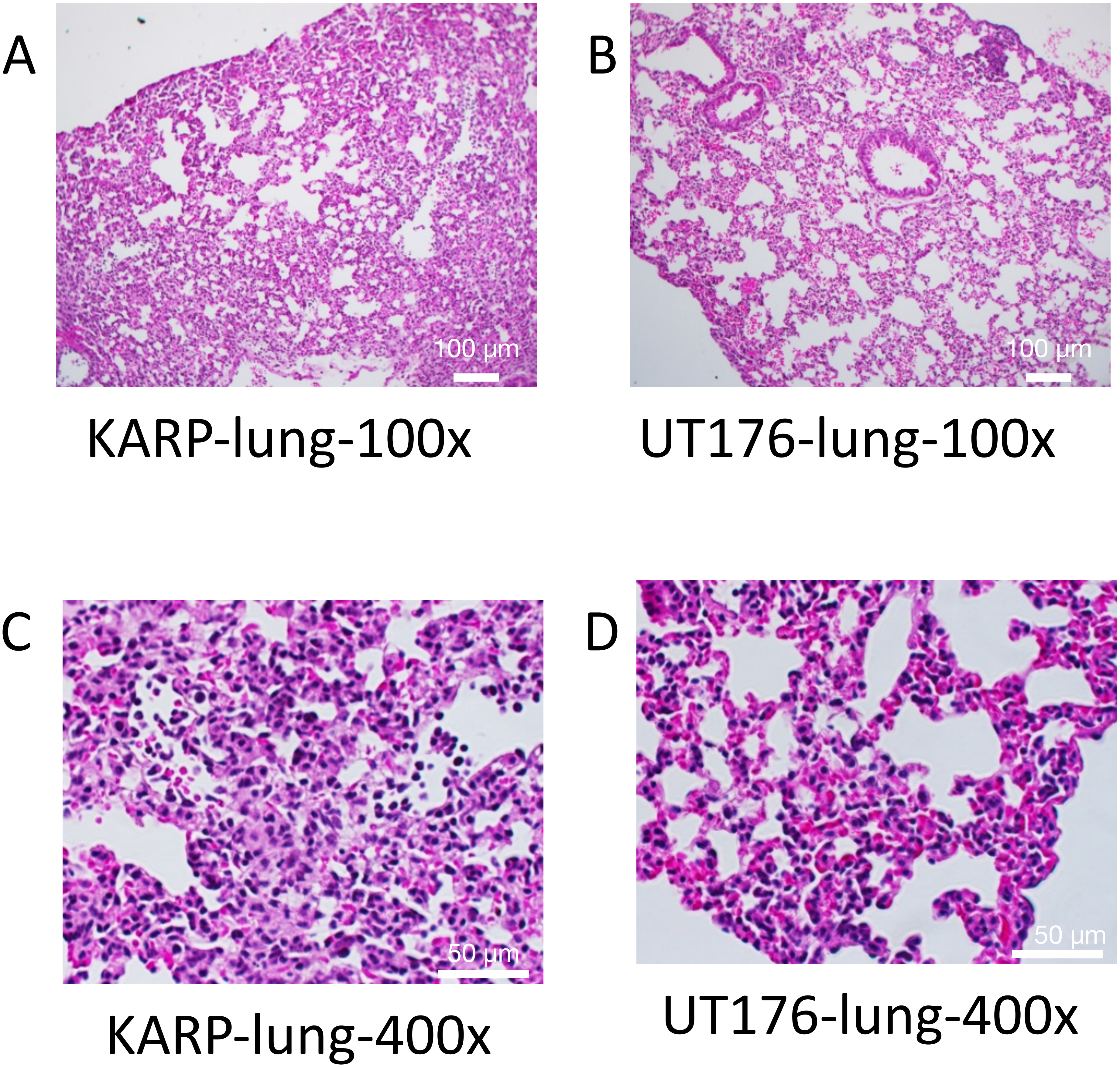

Supplementary Figure 19. Histopathological analysis of Karp- and UT176-infected mouse tissues. A-D. Karp-infected mouse lungs show increased cellular infiltration, compared with UT176 infected counterpart. E-I. Karp-infected mouse liver shows increased necrosis and inflammation, compared with UT176 infected counterpart. Arrows in E&I: boundaries of necrotic (nec) zone; Arrows in F&J: bigger and multiple inflammatory foci with cellular aggregation; Arrows in H: smaller and fewer inflammatory foci with cellular aggregation; Arrows in K: perivascular inflammatory foci. Figs I-L are magnified section of E-H, respectively. Samples prepared and imaged from 8 independent animals, representative images from one animal are shown here.

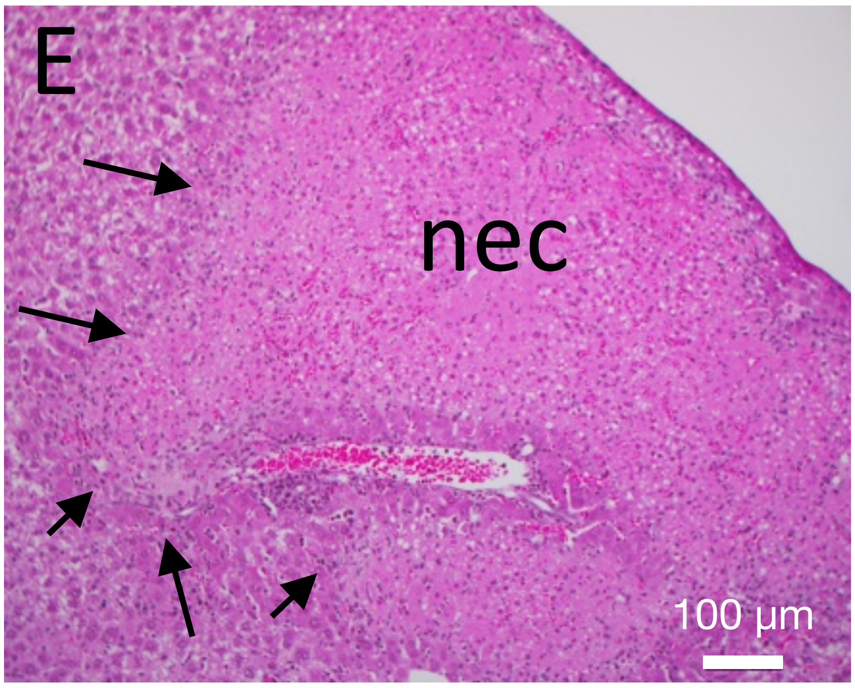

KARP-liver-100x-i

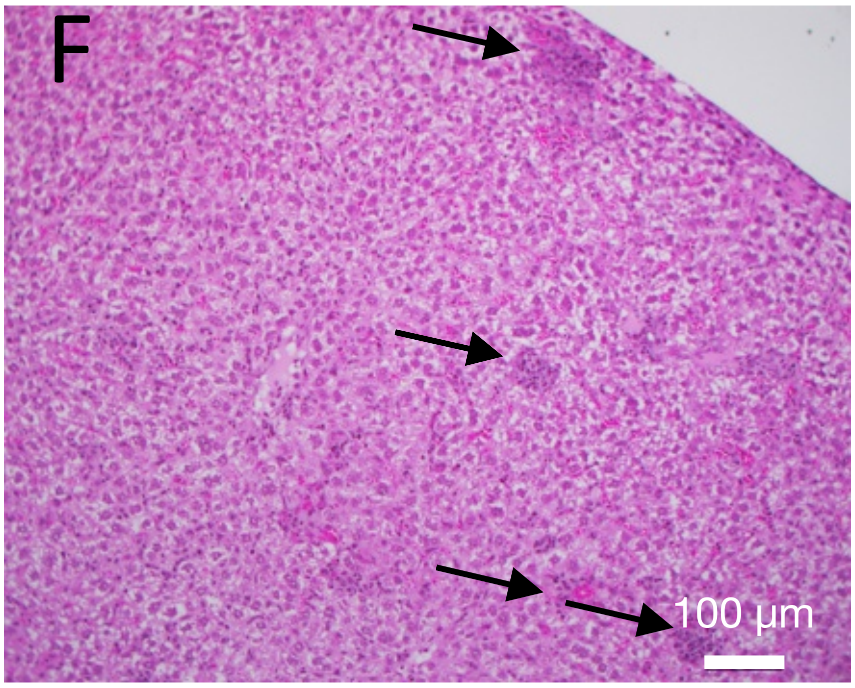

KARP-liver-100x-ii

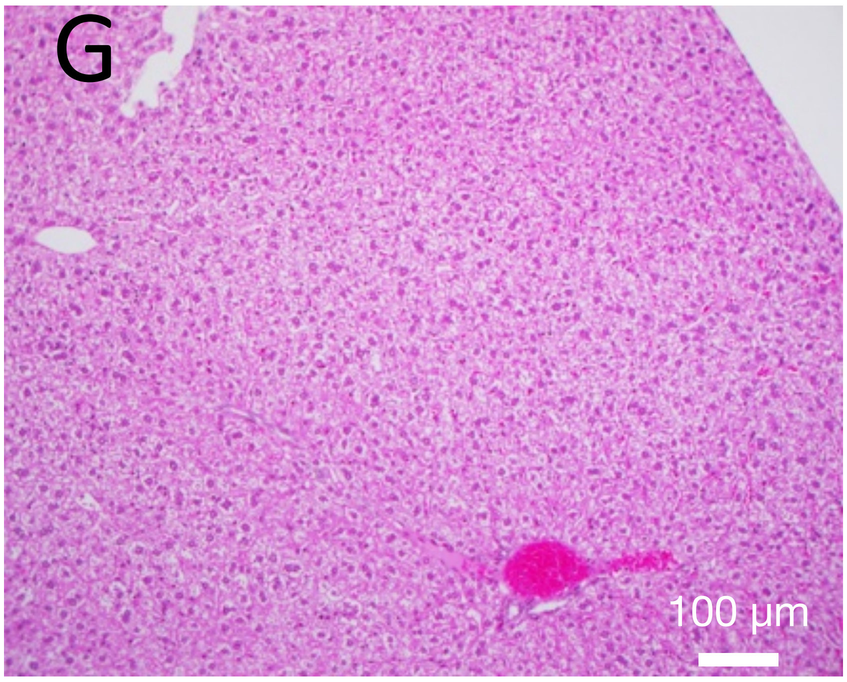

UT176-liver-100x-i

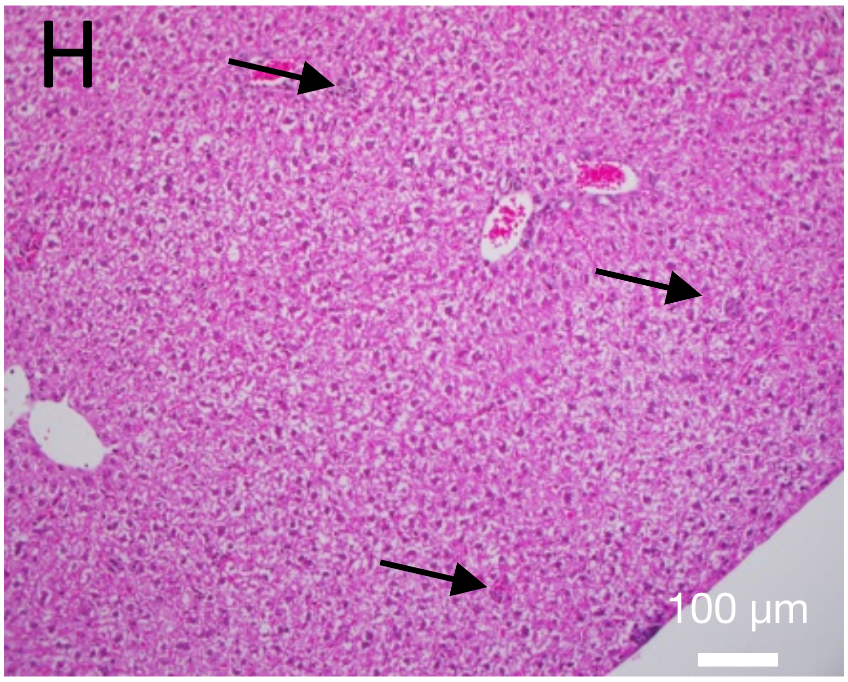

UT176-liver-100x-ii

Supplementary Figure 19. continued

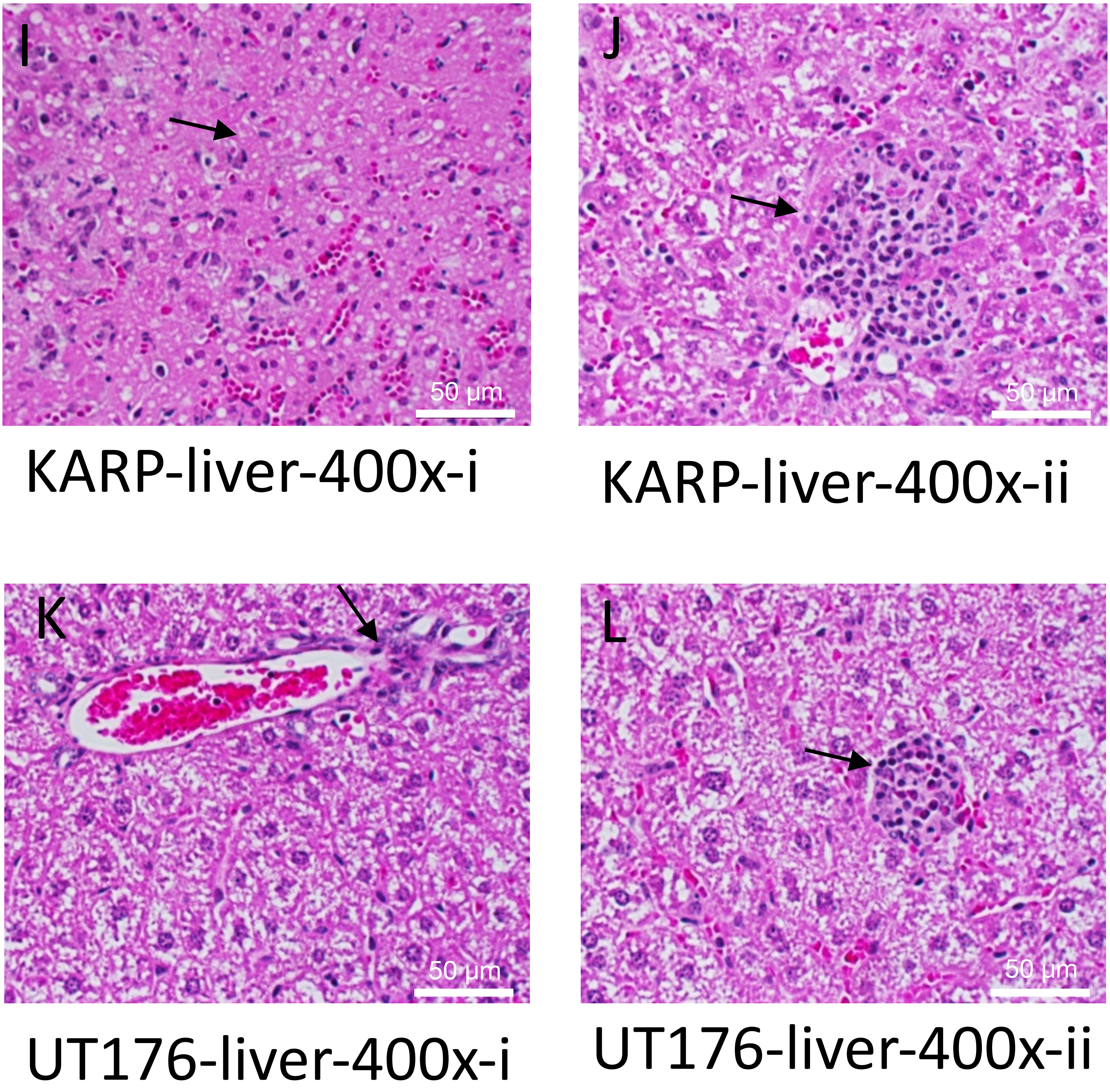

Supplementary Figure 19. continued

Northern blot probes	name	sequence (5'->3')	length [nt]	description	reference
	AWO-009	TACCTCTATTCTTAATAAAACTTATTGCC	29	antisense Northern probe against Orientia tmRNA (5')	this study
	AWO-010	TGATTTCCTTAAGCTGCTAATG	22	antisense Northern probe against Orientia tmRNA (3')	this study
	AWO-011	GGACTTTCCTCACAAATCTAT	21	antisense Northern probe against Orientia RNaseP RNA	this study
	AWO-013	GTTGATGCCTACGCCAGTTA	20	antisense Northern probe against Orientia SRP RNA	this study
	AWO-022	CTCTCCCATGTTTAAACATA	20	antisense Northern probe against Orientia 5S rRNA	this study
	JVO-7672	ATATGGAACGCTTCACGAATTTG	23	antisense Northern probe against human U6 snRNA	PMID:26789254
**qRT-PCR primers**	**name**	**sequence (5'->3')**	**length [nt]**	**description**	**reference**
	JVO-8896	TCGGTACATCCTCGACGG	18	sense qPCR primer against human IL6 mRNA	this study
	JVO-8897	TGTTTTCTGCCAGTGCCTC	19	antisense qPCR primer against human IL6 mRNA	this study
	JVO-14331	GCTGTGAAGATACGGGAGAGAAC	23	sense qPCR primer against human TNFAIP3 mRNA	this study
	JVO-14332	CCTGGATGTTTCTGTCGATGAG	22	antisense qPCR primer against human TNFAIP3 mRNA	this study
	JVO-9476	AAGTGGCTATGCTCAAAATG	20	sense qPCR against human IL7R mRNA	PMID:21307942
	JVO-9477	TTCAGGCACTTTACCTCCAC	20	antisense qPCR against human IL7R mRNA	PMID:21307942
	AWO-007	CAAACGATAGGCTCAAACACT	22	sense qRT-PCR oligo for human IL33 mRNA	this study
	AWO-008	TGAGTGTTGCCTAAGACATC	20	antisense qRT-PCR oligo for human IL33 mRNA	this study
	JVO-13531	ATGCAGGAAGAACATGACAACC	22	sense qRT-PCR oligo for human IFIT1 mRNA	this study
	JVO-13532	TCTGGACACTCCATTCTATAGCG	23	antisense qRT-PCR oligo for human IFIT1 mRNA	this study
	JVO-7673	GCTTCGGCAGCACATATACTAAAAT	25	sense qPCR primer against human U6 snRNA	PMID:26789254
	JVO-7672	ATATGGAACGCTTCACGAATTTG	23	antisense qPCR primer against human U6 snRNA	PMID:26789254
**RT-PCR primers**	**name**	**sequence (5'->3')**	**length [nt]**	**description**	**reference**
	47kda FW	TCCAGAATTAAATGAGAATTTAGGAC	26	Amplification of 47kda gene in Ot for bacterial quantification	PMID: 26317517
	47 kda RV	TTAGTAATTACATCTCCAGGAGCAA	25	Amplification of 47kda gene in Ot for bacterial quantification	PMID: 26317517
	47 kda PROBE	FAM-TTCCACATTGTGCTGCAGATCCTTC-	25	Amplification of 47kda gene in Ot for bacterial quantification	PMID: 26317517

Supplementary Table 1. Summary of primers and probes used in this study

Supplementary Discussion: Codon bias in Ot

A comparison of our RNAseq and proteomics datasets revealed core genes detected by RNAseq but not proteomics. Some of this discrepancy is likely due to differences in sample preparation and sensitivities between the two methods, loss of secreted proteins, and differences in protein stability. However, some of this difference might also be explained by differences in codon usage resulting in differences in translation efficiency. To address this question, we analyzed global codon usage in Ot and compared it with two other alpha-proteobacterial species, *Caulobacter crescentus* and *Rickettsia typhi* (Supplementary Figure 8A), and the gamma-proteobacterium *E. coli*. Genome-wide biases in Ot and *R. typhi* were very similar, but they differed from those in *C. crescentus* and *E. coli,* supporting previous analyses suggesting that codon bias in obligate intracellular bacteria is not strong, which may reflect slow growth rates not limited by the speed of translation[1–3]. We observed no clear correlation between codon usage and the presence or absence of a cognate tRNA in Ot. Ot encodes 34 out of 61 possible amino-acid coding tRNAs, leaving 27 potential codons without a cognate tRNA molecule. Of the 20 amino acids, 7 have a strong bias against the cognate Ot tRNA, 10 have biases for both cognate and non-cognate Ot tRNAs and only 3 have biases for the cognate Ot tRNA (Supplementary Figure 8, 9), unlike codon biases in other organisms, which correlate with tRNA copies[4–7]. There may be mechanisms by which bias towards non-cognate tRNA is preferential, or perhaps fast translation rates are not strongly selected for in Ot.

To determine whether Ot uses codon bias to regulate gene expression we compared codon usage between different groups of Ot genes (Supplementary Figure 8B, C). When comparing core and non-core gene groups, seventeen out of twenty amino acids showed differences in codon usage (Supplementary Figure 8B, 9), likely reflecting the horizontal acquisition of RAGE genes and possible differences in their subsequent optimization of translation.

We compared codon usage between core genes that were either detected or not detected in our proteomics dataset and found nine amino acids that were differentially encoded between these groups (Ala, Cys, Gly, Ile, Pro, Arg, Ser, Thr, Val) (Supplementary Figure 8B and C, Supplementary Figure 9). The differences were statistically significant but smaller than those seen in other organisms, such as *E. coli*[8,9]. We compared these codon usage biases to those of a group of seven genes whose expression was high based on relative peptide levels in the proteomics dataset (RpoB1, RpoB2, GroEL, DnaK, GdhA, HtpG, TSA56) and observed the same trend as seen between those not detected and detected in the proteomics dataset. Thus, these differences likely reflect a true relationship between codon usage and translation, although the contribution of this bias to protein expression remains unknown.

Supplementary References:

1. Andersson, S. G. & Sharp, P. M. Codon usage and base composition in Rickettsia prowazekii. *J Mol Evol* **42**, 525-536 (1996).
2. Botzman, M. & Margalit, H. Variation in global codon usage bias among prokaryotic organisms is associated with their lifestyles. *Genome Biol* **12**, R109 (2011).
3. Sharp, P. M., Bailes, E., Grocock, R. J., Peden, J. F. & Sockett, R. E. Variation in the strength of selected codon usage bias among bacteria. *Nucleic Acids Res* **33**, 1141-1153 (2005).
4. Duret, L. tRNA gene number and codon usage in the C. elegans genome are co-adapted for optimal translation of highly expressed genes. *Trends Genet* **16**, 287-289 (2000).
5. Ikemura, T. Correlation between the abundance of Escherichia coli transfer RNAs and the occurrence of the respective codons in its protein genes: a proposal for a synonymous codon choice that is optimal for the E. coli translational system. *J Mol Biol* **151**, 389-409 (1981).
6. Ikemura, T. Correlation between the abundance of yeast transfer RNAs and the occurrence of the respective codons in protein genes. Differences in synonymous codon choice patterns of yeast and Escherichia coli with reference to the abundance of isoaccepting transfer RNAs. *J Mol Biol* **158**, 573-597 (1982).
7. Plotkin, J. B. & Kudla, G. Synonymous but not the same: the causes and consequences of codon bias. *Nat Rev Genet* **12**, 32-42 (2011).
8. Frumkin, I., Lajoie, M. J., Gregg, C. J., Hornung, G., Church, G. M. & Pilpel, Y. Codon usage of highly expressed genes affects proteome-wide translation efficiency. *Proc Natl Acad Sci U S A* **115**, E4940-E4949 (2018).
9. Goodman, D. B., Church, G. M. & Kosuri, S. Causes and effects of N-terminal codon bias in bacterial genes. *Science* **342**, 475-479 (2013).
