# Supplementary material for: Dual RNA-seq of Orientia tsutsugamushi informs on host-pathogen interactions for this neglected intracellular human pathogen
Source: Nat Commun. 2020 Jul 3;11:3363. doi: 10.1038/s41467-020-17094-8 (PMC7335160; doi:10.1038/s41467-020-17094-8)
Supplement: Supplementary file 2 — Reporting Summary [file 41467_2020_17094_MOESM2_ESM.pdf]

nature research

Corresponding author(s): Jeanne Salje and Lars Barquist

Last updated by author(s): 2020/06/11

Reporting Summary

Nature Research wishes to improve the reproducibility of the work that we publish. This form provides structure for consistency and transparency in reporting. For further information on Nature Research policies, see Authors & Referees and the Editorial Policy Checklist .

Statistics

For all statistical analyses, confirm that the following items are present in the figure legend, table legend, main text, or Methods section.

n/a	Confirmed	
☐	☒	The exact sample size (*n*) for each experimental group/condition, given as a discrete number and unit of measurement
☐	☒	A statement on whether measurements were taken from distinct samples or whether the same sample was measured repeatedly
☐	☒	The statistical test(s) used AND whether they are one- or two-sided<br>*Only common tests should be described solely by name; describe more complex techniques in the Methods section.*
☐	☒	A description of all covariates tested
☐	☒	A description of any assumptions or corrections, such as tests of normality and adjustment for multiple comparisons
☐	☒	A full description of the statistical parameters including central tendency (e.g. means) or other basic estimates (e.g. regression coefficient) AND variation (e.g. standard deviation) or associated estimates of uncertainty (e.g. confidence intervals)
☐	☒	For null hypothesis testing, the test statistic (e.g. *F*, *t*, *r*) with confidence intervals, effect sizes, degrees of freedom and *P* value noted<br>*Give P values as exact values whenever suitable.*
☒	☐	For Bayesian analysis, information on the choice of priors and Markov chain Monte Carlo settings
☒	☐	For hierarchical and complex designs, identification of the appropriate level for tests and full reporting of outcomes
☐	☒	Estimates of effect sizes (e.g. Cohen's *d*, Pearson's *r*), indicating how they were calculated

Our web collection on statistics for biologists contains articles on many of the points above.

Software and code

Policy information about availability of computer code

Data collection	No software was used in data collection in this study.
Data analysis	All software used was either open source or commercially available. For RNA-seq data, cutadapt was used for read trimming, then reads were mapped with READemption (v0.4.3) / segemehl and the lack remapper (v0.2.0). Reads mapping to the bacterial genome were requantified using Salmon (v0.9.1) . Gene annotation was performed using eggNOG-mapper, and KEGGREST (v 1.18.1) and GO.db (v 3.5.0) were used to retrieve relevant functional terms. Non-coding RNAs were predicted using Rockhopper (v. 2.0.3), ANNOgesic (v0.7.17) , and Infernal (v1.1.2), and visualized with the Integrative Genomics Viewer (v2.5.2). Gene synteny plots were drawn using EasyFig. Orthology relationships between bacterial genomes were predicted using Poff (included in ProteinOrtho v 5.16). Differential gene expression analysis was performed with edgeR (v3.20.9) . Proteomics data were quantified using MaxQuant (v1.5.5.1). The caret package in R was used for training and testing logistic regression models, and the pROC package was used for determining ROC curves. Pathways analysis was performed using the fry function in the edgeR R package for the bacteria, and the Ingenuity Pathway Analysis tool for host cells. E. coli protein abundance was retrieved from PaxDB. Student's t-test for infection assays was performed in GraphPad Prism (v.8.3.0)

For manuscripts utilizing custom algorithms or software that are central to the research but not yet described in published literature, software must be made available to editors/reviewers. We strongly encourage code deposition in a community repository (e.g. GitHub). See the Nature Research guidelines for submitting code & software for further information.

Data

Policy information about availability of data

All manuscripts must include a data availability statement. This statement should provide the following information, where applicable:

- Accession codes, unique identifiers, or web links for publicly available datasets
- A list of figures that have associated raw data
- A description of any restrictions on data availability

Sequencing data has been deposited in GEO with accession number GSE139498. Proteomics data has been deposited in PRIDE with accession number XXXXXX.

Field-specific reporting

Please select the one below that is the best fit for your research. If you are not sure, read the appropriate sections before making your selection.

☒ Life sciences ☐ Behavioural & social sciences ☐ Ecological, evolutionary & environmental sciences

For a reference copy of the document with all sections, see nature.com/documents/nr-reporting-summary-flat.pdf

Life sciences study design

All studies must disclose on these points even when the disclosure is negative.

Sample size	No power analysis was undertaken to determine sample size. We used a sample size of 3, as previous work has suggested this is adequate to detect differential expression in transcripts with a large fold-change (see Westermann et al., "Dual RNA-seq unveils noncoding RNA functions in host-pathogen interactions", Nature 2016)
Data exclusions	No data was excluded.
Replication	We performed qRT-PCR in independent infection samples to verify differential expression. These confirmed the direction of differential expression predicted by RNA-seq. Experiments were performed in triplicate, and data from all experiments performed is shown in Supplementary Figure 12.
Randomization	Randomization was not used, as experiments involved pure cultures of genetically identical organisms.
Blinding	Blinding was not possible, as the correct reference genomes had to be used in analysis of each sample.

Reporting for specific materials, systems and methods

We require information from authors about some types of materials, experimental systems and methods used in many studies. Here, indicate whether each material, system or method listed is relevant to your study. If you are not sure if a list item applies to your research, read the appropriate section before selecting a response.

Materials & experimental systems

n/a	Involved in the study	
☒	☐	Antibodies
☐	☒	Eukaryotic cell lines
☒	☐	Palaeontology
☐	☒	Animals and other organisms
☒	☐	Human research participants
☒	☐	Clinical data

Methods

n/a	Involved in the study	
☒	☐	ChIP-seq
☒	☐	Flow cytometry
☒	☐	MRI-based neuroimaging

Eukaryotic cell lines

Policy information about cell lines

Cell line source(s)	HUVEC, Gibco catalog number C0035C, lot number 1702784; L929 source Dr. Stuart Blacksell Mahidol Oxford Tropical Medicine Research Unit, Bangkok, Thailand
Authentication	Not authenticated
Mycoplasma contamination	Tested negative for mycoplasma contamination
Commonly misidentified lines (See ICLAC register)	No commonly misidentified cell lines were used in this study

Animals and other organisms

Policy information about studies involving animals; ARRIVE guidelines recommended for reporting animal research

Laboratory animals	Mus musculus strain C57BL/6NJcl aged 6-8 weeks, male and female
Wild animals	The study did not involve wild animals
Field-collected samples	The study did not involve animals collected from the field
Ethics oversight	IACUC and Biosafety Review Committee at the Armed Forces Research Institute of Medical Sciences (AFRIMS) Bangkok, Thailand

Note that full information on the approval of the study protocol must also be provided in the manuscript.
