# Supplementary material for: Dual RNA-seq of Orientia tsutsugamushi informs on host-pathogen interactions for this neglected intracellular human pathogen
Source: Nat Commun. 2020 Jul 3;11:3363. doi: 10.1038/s41467-020-17094-8 (PMC7335160; doi:10.1038/s41467-020-17094-8)
Supplement: Supplementary file 3 — Description of Additional Supplementary Files [file 41467_2020_17094_MOESM3_ESM.pdf]

# Description of Additional Supplementary Files

Supplementary Data 1: Mapping statistics

This sheet shows percentage of RNA-seq reads assigned to different classes of RNA in Karp, UT176 and HUVEC.

Supplementary Data 2: Conserved islands

This sheet describes conserved islands identified in Batty et al 2018.

Supplementary Data 3: Conserved operons

This sheet shows conserved operons identified in both Karp and UT176.

Supplementary Data 4: Karp specific operons

This sheet shows operons identified only in Karp.

Supplementary Data 5: UT176 specific operons

This sheet shows operons identified only in UT176.

Supplementary Data 6: Joint response

This sheet shows genes upregulated (logFC > 2.0 and FDR < 0.01) in Karp- and UT176-infected HUVEC cells compared with uninfected HUVEC cells.

Supplementary Data 7: Host differential expression long list

This sheet describes host genes differentially expressed by HUVEC in response to Karp or UT176.

Supplementary Data 8: Host differential expression short list

This sheet describes selected host genes differentially expressed by HUVEC in response to UT176 or Karp and uninfected HUVEC cells.

Supplementary Data 9: Core genes

This sheet includes a list of core genes identified in Batty et al 2018 and their presence or absence in our proteomics dataset.

Supplementary Data 10: Karp groups of duplicates

This sheet describes the groups of the Karp genes identified as duplicates by Salmon.

Supplementary Data 11: UT176 groups of duplicates

This sheet describes the groups of the UT176 genes identified as duplicates by Salmon.

Supplementary Data 12: Karp expressed

This sheet lists all genes expressed in Karp strain grown in HUVEC cells. Expressed is defined as having TPM mean values from 3 replicates greater than 10.

Supplementary Data 13: Karp highly expressed

This sheet lists all genes highly expressed in Karp strain grown in HUVEC cells. Highly expressed is defined as having TPM mean values from 3 replicates greater than 50.

Supplementary Data 14: Karp all genes summary

This sheet lists all quantified genes in Karp strain grown in HUVEC cells.

Supplementary Data 15: UT176 all genes summary

This sheet lists all quantified genes in UT176 strain grown in HUVEC cells.

Supplementary Data 16: Bacterial differential expression long list

This sheet describes results of differential expression of bacterial genes in HUVEC cells infected with UT176 or Karp. UT176 gene expression was compared to Karp gene expression. Higher logFC indicates higher expression of genes in UT176 than in Karp.

Supplementary Data 17: Bacterial differential expression short list

This sheet describes results of differential expression of bacterial genes in HUVEC cells infected with UT176 or Karp. UT176 gene expression was compared to Karp gene expression.

Supplementary Data 18: Bacterial differential expression Ank TPR

This sheet describes results of the differential expression of ankyrin-repeat and TPR-repeat bacterial genes in HUVEC cells infected with UT176 or Karp. UT176 gene expression was compared to Karp gene expression.

Supplementary Data 19: Gene set enrichment analysis

This sheet describes results of FRY gene set analysis which shows an enrichment of the gene sets in differently expressed genes between UT176 and Karp infecting HUVEC cells.

Supplementary Data 20: Proteomics

This sheet contains proteomics data of both human and Karp.

Supplementary Data 21: Predicted ncRNA Karp

This sheet describes genome coordinates of predicted ncRNAs in Karp.

Supplementary Data 22: Predicted ncRNA UT176

This sheet describes genome coordinates of predicted ncRNAs in UT176.
